# Self-organized formation of developing appendages from murine pluripotent stem cells

**DOI:** 10.1038/s41467-019-11702-y

**Published:** 2019-08-23

**Authors:** Shunsuke Mori, Eriko Sakakura, Yuji Tsunekawa, Masaya Hagiwara, Takayuki Suzuki, Mototsugu Eiraku

**Affiliations:** 1 Laboratory of Developmental Systems, Institute for Frontier Life and Medical Sciences, Kyoto University, Kyoto, 606-8507 Japan; 2 Laboratory for in vitro Histogenesis, RIKEN Center for Developmental Biology, Kobe, 650-0047 Japan; 3 Department of Genetics, Rutgers, The State University of New Jersey, Piscataway, NJ 08854 USA; 4 Laboratory for Cell Asymmetry, RIKEN Center for Developmental Biology, Kobe, 650-0047 Japan; 5 NanoSqure Research Institute, Osaka Prefecture University, Osaka, 599-8570 Japan; 6 Laboratory of Avian Bioscience, Graduate School of Bioagricultural Sciences, Nagoya University, Nagoya, 464-8602 Japan; 7 Institute for Advanced Study of Human Biology (WPI-ASHBi), Kyoto University, Yoshida-Konoe-cho, Sakyo-ku, Kyoto 606-8501 Japan

**Keywords:** Limb development, Stem-cell differentiation

## Abstract

Limb development starts with the formation of limb buds (LBs), which consist of tissues from two different germ layers; the lateral plate mesoderm-derived mesenchyme and ectoderm-derived surface epithelium. Here, we report means for induction of an LB-like mesenchymal/epithelial complex tissues from murine pluripotent stem cells (PSCs) in vitro. The LB-like tissues selectively differentiate into forelimb- or hindlimb-type mesenchymes, depending on a concentration of retinoic acid. Comparative transcriptome analysis reveals that the LB-like tissues show similar gene expression pattern to that seen in LBs. We also show that manipulating BMP signaling enables us to induce a thickened epithelial structure similar to the apical ectodermal ridge. Finally, we demonstrate that the induced tissues can contribute to endogenous digit tissue after transplantation. This PSC technology offers a first step for creating an artificial limb bud in culture and might open the door to inducing other mesenchymal/epithelial complex tissues from PSCs.

## Abstract (web summary)

Organoid technology has been applied to model many types of organs in vitro, although structures containing multiple germ layers, such as limb buds, have not been generated. Here, the authors induce limb bud-like tissues from mouse ESCs that can be functionally integrated after transplantation.

## Introduction

A three-dimensional (3D) culture of PSCs has been reported to enable the formation of organoids with various well-recapitulated aspects of organogenesis, such as tissue patterning, morphogenesis, proper arrangement of each cell type, and developmental timing^1^. Recently, in vitro formation of organoids for various tissues including the brain^2^, retina^3^, intestine^4^, and kidney^5^ has been actively studied, whereas, in vitro induction of trunk appendage organoids such as limb bud (LB), in which mesenchymal cells are covered with a surface epithelial sheet, has not yet been reported.

LB mesenchyme is derived from the lateral plate mesoderm (LPM). The mesodermal specification could be correlated with the anterior–posterior patterning of the primitive streak. LPM is derived from the posterior part of the primitive streak and gives rise to LB mesenchyme, branchial arch mesenchyme and cardiac mesoderm^6^. In the mouse embryo, the forelimbs form at the level of somite 7–12 and the hindlimbs at somite 23–28. Specification of limb-type occurs prior to the initiation of LB outgrowth under the regulation of an axial Hox code^7,8^. At a later stage of development, LBs grow in the distal direction and form orderly patterned digits. Both LB outgrowth and digit patterning require the formation of two signaling centers in LB; the apical ectodermal ridge (AER) and zone of polarizing activity (ZPA). AER is located at the tip of the LB and is involved in promoting limb outgrowth and in coordinating proximal–distal patterning. Signaling along the anterior–posterior axis is organized by the ZPA which is localized to the posterior margin of the LB. Both signaling centers coordinate the LB axial patterning through secreted signaling factors^9^.

In this study, we report a means for the induction of forelimb bud (FLB) and hindlimb bud (HLB)-like tissues from mouse embryonic stem cells (mESCs). We also demonstrate that the surface ectoderm in mESC-derived LBs (ES–LBs) forms an AER-ES-like structure in response to an artificially manipulated BMP signaling, and ES–LBs were functionally integrated after transplantation into the developing mouse limbs.

## Results

### Induction of hand2-positive LPM-like tissue from mouse ESCs

**Fig. 1:**
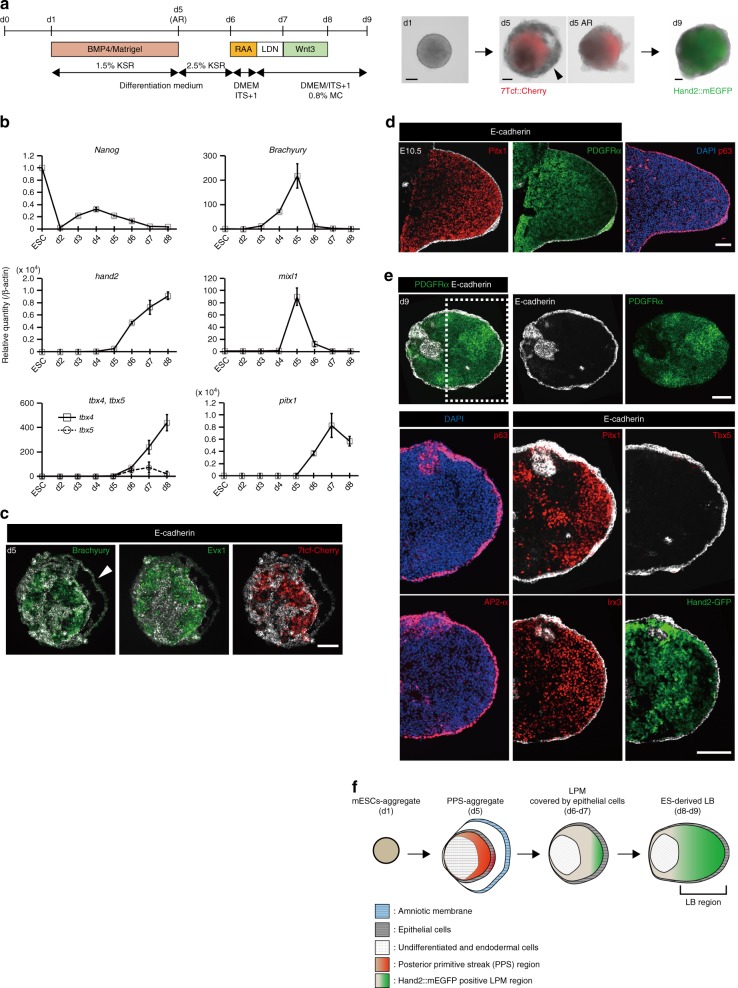
Self-formation of a hindlimb bud-like tissue in 3D culture of mESCs aggregate. **a** Scheme for induction of HLB from mESCs aggregate (left), and bright-field views (right). **b** qRT-PCR analysis of each marker in ES–HLB from day 2 to 8. (mean ± s.d, *n* = 3 independent experiments). **c** Cryosection of the mESC-derived PPS aggregate on day 5 was stained with E-cadherin, Brachyury, Evx1, and fluorescence of 7Tcf::Cherry. **d**, **e** Immunostaining of D-V section of the E10.5 mouse hindlimb bud **d** and ES–HLB on day 9 (e; top, whole ES–HLB section; bottom, enlarged view of dotted box area). Hindlimb bud mesenchymal cells (Pitx1+, PDGFRα+) are covered by the epithelial layer (E-cadherin+, p63+, AP2-α+). Forelimb marker (Tbx5) is not expressed, and A-P markers (A: Irx3, P: Hand2::mEGFP) are expressed in ES–HLB. **f** Schematic differentiation process of ES–LB from mESCs aggregate. AR, amniotic membrane removed; LPM, lateral plate mesoderm; D-V, dorsal-ventral; A-P, anterior–posterior; mESCs, mouse embryonic stem cells; ES–HLB, mESC-derived hindlimb bud. Arrowheads indicate amniotic membrane. Scale bars, 100 μm

For in vitro induction of LB-like tissues from PSCs, we first modified the optic cup induction method namely; serum-free floating culture of embryoid body-like aggregate with quick reaggregation (SFEBq)^3^. During embryonic gastrulation, the posterior primitive streak (PPS) gives rise to the LPM and intermediate mesoderm (IM). The mesodermal positional values along the mediolateral axis (LPM or IM) are controlled by the signal intensity of bone morphogenetic protein (BMP)^10^. BMP signaling also has a role in the induction of the primitive streak and the non-neural ectoderm^11,12^. Therefore, we tested the effect of BMP4 treatment on optic cup induction. To monitor the differentiation state of mESC aggregates, we generated a mESC line with dual reporter fluorescence; mCherry regulated under the 7Tcf promoter^13^ and EGFP knocked-in a *hand2* gene locus (Supplementary Fig. 1). Using this cell line, both Wnt signaling (7Tcf activity)^14^ and LPM induction (Hand2 expression)^15^ could be simultaneously monitored. In the presence of BMP4 and Matrigel (days 1–5), mESC aggregates differentiated into tissues with the outer epithelial layer and the inner cell aggregate on day 3 (Supplementary Fig 2a). Both Brachyury (also known as T) and 7Tcf::Cherry began to be expressed in a polarized manner within the inner cell aggregate on day 4 (Fig. 1a, b, Supplementary Fig 2a, Supplementary Movie 1). Immunostaining and qPCR analysis of the FACS-sorted cells revealed that 7Tcf::Cherry-positive cells expressed PPS markers, Wnt3, T, Mixl1 and Evx1 on day 5 (Fig. 1b, c and Supplementary Fig. 2b–d)^16^. On day 5, very few populations of cells marked with each of the pluripotent (Oct3/4, Nanog), endodermal (FoxA2, Sox17) and neuroepithelial (Sox1) marker genes were also detected in the 7Tcf-negative (7Tcf^−^) region (Supplementary Fig. 2d, e). Furthermore, we noticed that the surface of 7Tcf-positive (7Tcf^+^) region was covered with an E-cadherin-positive layer that partially co-expressed the epithelial-mesenchymal transition markers N-cadherin and Snail, which is a hallmark of gastrulation^17^ (Supplementary Fig. 2f). On the other hand, until day 5 of culture, the outermost thin layer expanded outward and formed a balloon-like structure in which cells showed intense staining with antibodies for Msx1/2, pSmad1/5, AP2-α, Oct3/4, and Laminin α1/β1, but not T (Supplementary Movie 1 and Supplementary Fig. 2a, e). We therefore assumed that the outermost thin layer was presumptive amniotic membrane^18^. To maintain the culture of the inner cells in a healthy state, this outermost structure had to be mechanically removed on day 5 (Fig. 1a and Supplementary movie 2). After removal of the presumptive amniotic membrane, the inner PPS-like tissues spontaneously differentiated into Hand2-positive LPM tissues until day 7 (Fig. 1b). Almost all mESC aggregates treated with BMP4 in the presence of Matrigel that we observed had differentiated into PPS (7Tcf^+^) and then into the LPM (Hand2^+^). This directional differentiation of PPS into LPM was specifically inhibited by a short-term treatment on day 5 with BMP inhibitors LDN193189 (LDN) or Dorsomorphin (DM), which changed the differentiation state from LPM to IM-like cells (Supplementary Fig. 3). In contrast, none of the inhibitors against Wnt, Shh, and activin had any effect (Supplementary Fig. 3b). These results suggest that the intensity of endogenous BMP signaling controls the positional values along the media-lateral axis of the mesoderm induced from PSCs, as seen in embryonic development.

### Self-organized formation of HLB-like tissues from SFEBq aggregates

In the developing mice embryo, Hand2-positive LPM gives rise to mesenchymal cells located in LBs as well as in the branchial arch and the heart. So, we next investigated the expression of transcriptional factors which are specifically expressed in developing LBs. We examined two T-box genes, Tbx5 and Tbx4, which are markers for the FLB and HLB, respectively^19^. On day 6 of culture, both Tbx4 and another HLB marker Pitx1 were detected in mESC-derived LPM tissues by immunostaining and qPCR, however, the FLB marker Tbx5 was not (Fig. 1b and Supplementary Fig. 4a), suggesting that the mESC-derived LPM specifically differentiates into tissues with characteristics of the HLB. We also noticed that transient treatment of a retinoic acid antagonist (RAA, 1 µM) on day 6 substantially increases Pitx1 expression in a dose-dependent manner (Supplementary Fig. 4b). RA signaling is also known to be involved in the early initiation of LBs^20^, consistent with this, higher concentrations of RAA (40 µM) suppressed the expression of Meis1/2, Hand2, and Tbx4 (Supplementary Fig. 4c). Until day 9, Pitx1-positive cells matured into mesenchymal cells expressing PDGFR (Fig. 1d, e). Interestingly, a composite structure in which Pitx1-positive mesenchymal cells were wrapped by an ectodermal layer (E-cadherin^+^, p63^+^, AP2-α^+^, Keratin8/18^+^, and Keratin5^+^) had been repeatedly observed (Fig. 1d, e, Supplementary Fig. 5a, b and Supplementary Movie 3). However, the epithelial layer on the surface was easily disintegrated and unstable, and it was frequently observed that mesenchymal cells protruded from the site which was not covered by the epithelial layer (Supplementary Fig. 5c). According to previous reports^21^, transient treatment of recombinant Wnt3 protein reinforced the epithelial stability (Supplementary Fig. 5c).

Formation of three different axes (P-D, A-P, and D-V) is required for LB elongation and pattern formation. So, we next focused on spontaneous patterning in ES–LBs. Irx3 and Hand2 that are anterior and posterior markers respectively, were expressed in the inner mesenchyme of ES–HLB in a mutually exclusive manner^15^, however, the distribution of cells expressing these markers showed no reproducible pattern (Fig. 1e, and Supplementary Fig 5a). Similarly, cells expressing the proximal markers, Meis1/2 and Pbx1/2, were uniformly distributed (Supplementary Fig. 5b). On the other hand, Msx1/2 expressed in the distal area of the LB was mainly detected in cells close to the layer of surface epithelium (Supplementary Fig. 5b). Thus, in the initial culture environment containing BMP4 and Matrigel, SFEBq aggregates self-organized HLB-like tissues consisting of LPM-derived mesenchymal cells enveloped by the epithelial tissue, however, the inner mesenchyme did not form axial patterns as seen in the developing LBs.

### Timed treatment of RA affects the positional value of ES–LBs

**Fig. 2:**
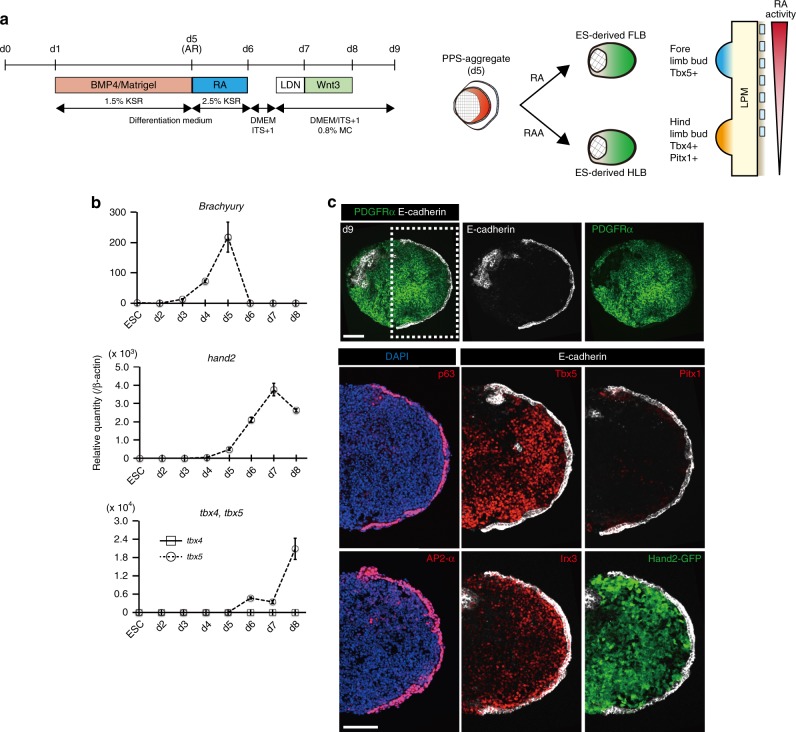
Retinoic acids induce a positional shift of ES–LB from hind to the forelimb. **a** Scheme for differentiation protocol of ES–FLB from mESCs aggregates (left). Schematic model of selective differentiation of fore or hindlimb bud from mESCs aggregate (right). **b** Time-course qRT-PCR analysis of ES–FLB from day 2 to 8. mESCs is a control group. (mean ± s.d, *n* = 3 independent experiments). **c** Immunostaining of ES–FLB on day 9 (top, whole ES–derived HLB section; bottom, enlarged view of dotted box area). Forelimb bud mesenchymal cells (Tbx5+, PDGFRα+) are covered by the epithelial layer (E-cadherin+, p63+, AP2-α+). Hindlimb marker (Pitx1) is not expressed, and A-P markers (A: Irx3, P: Hand2::mEGFP) are expressed in ES–FLB cells. AR, amnione-like membrane removal; LDN, LDN193189 (BMP inhibitor); LPM, lateral plate mesoderm; RA, retinoic acid; RAA, retionoic acid antagonist; ES–FLB, mESCs-derived forelimb bud. Scale bar, 100 μm

We next tried to induce FLB-like tissues from mESC aggregates by manipulating positional cues of the trunk mesoderm. The vertebrate embryo forms rostral-caudal gradients of high retinoic acid concentrations (RA, rostral) to high fibroblast growth factors (FGFs, caudal) for trunk mesodermal patterning^22^. It has been reported that the positional values can be manipulated in PSC-derived organoids by adjusting the concentration of RA and FGFs^5^. Furthermore, the above results, in which RA antagonists increased the expression of Pitx1 in ES–HLB cultures, suggested that RA signaling might be involved in rostral-caudal patterning of the induced LPM in vitro. We therefore tested transient treatment of the ES–LB culture with RA from day 5 to 6 (Fig. 2a). As expected, expression of the FLB marker Tbx5 markedly increased in ES–derived LPM aggregates during day 5–6, depending on RA concentration, whereas that of the HLB markers decreased (Fig. 2b, Supplementary Fig. 4a, b). Thus, transient treatment of RA could shift the induced mesenchyme into FLB-like, though the tissue structure wrapped in the surface epithelium and the expression pattern of axial markers were the same as those observed in ES–HLB (Fig. 2c and Supplementary Fig. 4 and 5b). We confirmed that similar results can be obtained using genetically altered mESC lines E14tg2a and 46C (Supplementary Fig. 6). These results indicate that the positional identity of ES–LBs could be manipulated by a dose and time-dependent RA signal.

### Comparative transcriptome analysis of ESC-derived LB-like tissues

**Fig. 3:**
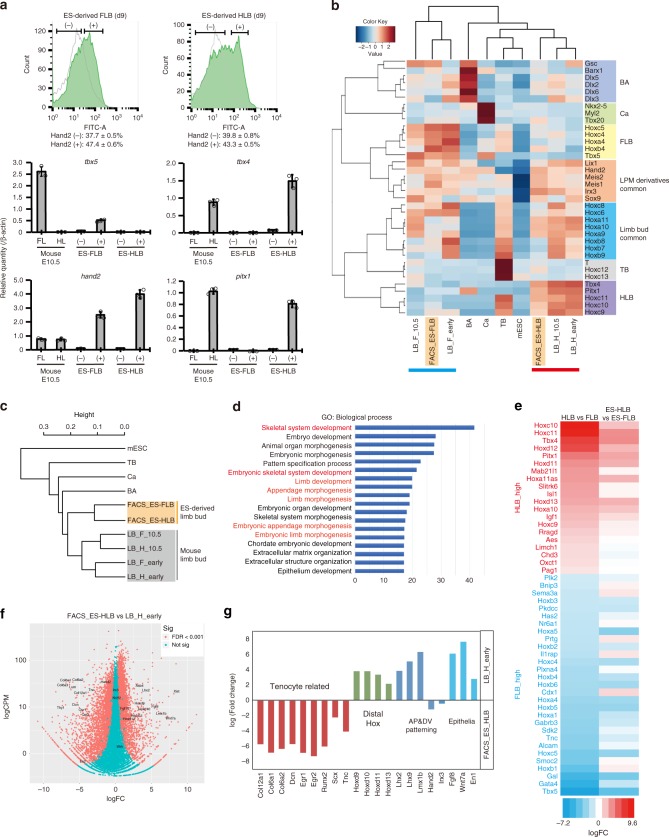
Comparative transcriptome analysis indicates similarity of ES–LB and in vivo limb bud. **a** FACS analysis of the ES–FLB (left) and -HLB (right) on day 5 (gray) and day 9 (green) (left side graphs). qRT-PCR analysis using FACS-sorted Hnad2::mEGFP-positive (+) and negative (−) cells. Gene expression levels are compared with E10.5 mouse fore and hindlimb buds (mean ± s.d, *n* = 3 independent experiments) (right side graphs). **b** Two-way clustering heat map visualizing each of tissue markers and region-specific Hox genes in ES–LB mesenchyme as compared with mouse embryonic tissues. mESC, mouse ES cells; Ca, cardiac; TB, tail bud; BA, branchial arch; LB_F and LB_H, mouse fore and hindlimb bud (F_early, E9.75; H_early, E10.25; 10.5, E10.5); FACS_ES–FLB and FACS_ES–HLB, FACS-sorted Hand2::mEGFP + mesenchyme form ES–derived FLB and HLB. **c** Hierarchical clustering of ES–LB mesenchyme with mouse embryonic tissues, based on gene ontology (GO:0048568)-recorded 485 genes set. **d** Biological process ontology. ToppGenes was utilized to perform functional enrichment of top 200 upregulated genes in the ES–HLB induction from mESC. **e** Differential gene expression analysis in FLB vs HLB and ES–FLB vs ES–HLB. Top 50 genes that showed the most different in DGE analysis (FLB vs HLB) were used. Almost genes show similar expression specificity in vitro and in vivo. **f** Volcano plot for HLB vs ES–HLB. **g** Genes showing different expression patterns in vitro and in vivo. ES–FLB and HLB, mESCs-derived fore and hindlimb bud

To further characterize the ES–LBs, we next performed a comparative transcriptome analysis using FACS-sorted Hand2^+^ mesenchymal cells from ES–LBs on day 9 (Fig. 3a and Supplementary Fig. 7a). The following tissues were prepared for comparison; embryonic LBs (forelimb; E9.75/E10.5 and hindlimb; E10.25/E10.5) and other E10.5 embryonic tissues (branchial arch, heart, and tail bud). By analyzing the regional markers that are specifically expressed in the developing cranial and trunk mesoderm^19,23^, it was confirmed that the positional identity of the ES–FLB and ES–HLB corresponds to the developing FLB and HLB, respectively (Fig. 3b). In particular, the *Hoxc* cluster genes show similar expression patterns in vivo and in vitro; *Hoxc4* and *Hoxc5* are highly expressed in both of FLBs and ES–FLBs, whereas *Hoxc9*, *Hoxc10,* and *Hoxc11* are only detected in both of HLBs and ES–HLBs. On the other hand, the cardiac mesoderm specific marker *Nkx2.5* and branchial arch specific genes such as *Dlx5/6* and *Barx1* were hardly expressed in ES–LBs. In addition, hierarchical clustering of transcriptional profiles using a 489 gene set associated with organ development (GO:0048568) showed that profiles of ES–LBs matched most closely to those of mouse LBs among these tissues (Fig. 3c). Furthermore, gene ontology (GO) analysis revealed that genes related with skeletal development and limb/appendage development are specifically upregulated during ESC differentiation (Fig. 3d). Differential gene expression (DGE) analysis also revealed that the difference in gene expression between forelimb and hindlimb resembles well in vivo and in vitro (Fig. 3e, Supplementary Fig. 8a). Altogether, these results clearly indicate that mESC aggregates treated with BMP4 in the presence of matrigel differentiated into the tissues that have a regional identity closer to that of LBs than other tissues derived from the trunk mesoderm. On the other hand, we also observed substantial differences in the LB mesenchyme in vitro and in vivo (Fig. 3f, g, Supplementary Fig 8c–e). First, DGE analysis unexpectedly revealed that, in the ES–LBs, genes such as *Col12a1, Col6a1, Tnc, Dcn, and Egr1*^24^, associated with tenogenic differentiation, showed higher expression levels than those seen in embryonic LBs (Fig. 3f, g). Second, as opposed to the proximal markers Pbx1/2 and Fgf10, the distal markers, including distal *hox* genes, such as *Hoxd9*, *Hoxd10* and *Hoxd11*, which contribute to distal outgrowth after formation of signaling centers (AER and ZPA) at a later stage^25^, showed lower expression in ES–LB tissues (Fig. 3g, Supplementary Fig. 7b and Supplementary Fig. 8c, e). These results imply that the ES–LB might be in the state of pre-outgrowth and non-patterned, owing to the absence of signals from signaling centers, such as the AER and ZPA. Consistent with this, as shown above, cells expressing proximal markers Meis1/2 and Pdx1/2 are uniformly distributed in ES–LBs without PD polarity (Figs. 1e, 2c and Supplementary Fig. 5b).

### Induction of AER-like tissue by manipulation of BMP signaling

**Fig. 4:**
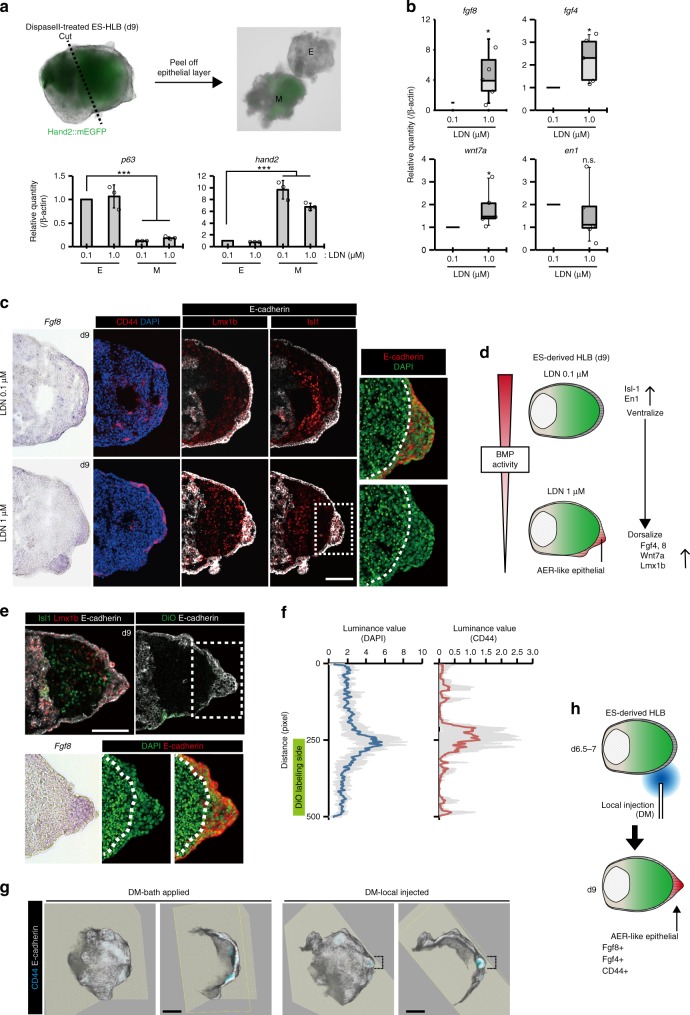
BMP inhibition induces AER-like structure via dorasalization of surface ectoderm. **a** Separation of the epithelial layer and mesenchyme aggregate from ES–HLB (upper). qRT-PCR analysis of gene expression of epidermis marker (p63) and LPM marker (hand2) (right, mean ± s.d, *n* = 3 independent experiments each 10 aggregates, ***P < 0.005 compared with LDN 0.1 μM epithelial samples; two-tailed Student’ s *t* test, lower). **b** The boxplot shows the qRT-PCR analysis of gene expression of ES–derived HLB epithelial layer. AER markers (fgf8 and fgf4) and D-V (wnt7a-en1) ectoderm markers expression level compares it between LDN 0.1 μM and 1 μM (mean ± s.d, *n* = 5 independent experiments each 10 aggregates, *P < 0.05 compared to LDN 0.1 μM; two-tailed Student’s *t* test). **c** Cryosection of LDN (0.1 and 1 μM) applied ES–derived HLB. Fgf8 (in situ) indicates AER-like epithelial layer. Immunostaining of Lmx1b-Isl1, E-cadherin, and CD44 are used as D-V, epithelial layer, and AER marker, respectively. DAPI/E-cadherin-staining in an enlarged view of dotted box area (dotted lines indicate basal side of epithelial layer). **d** Schematic model of the induction of AER-like tissue in an LDN dose-dependent manner. **e** Cryosection of ES–HLB locally applied with DM. Lmx1b-Isl1 uses as D-V marker. The injected point was labeled by DiO. Fgf8 (in situ) and DAPI/E-cadherin-staining in an enlarged view of dotted box area (dotted lines indicate the basal side of the epithelial layer). **f** Quantitative analysis in thickness of the epithelial layer (left, blue line) and CD44-expressed region (right, red line) in ES–HLB locally applied with DM. (light gray, mean ± s.d, *n* = 6 independent experiments). **g** 3D reconstruction of ES–HLB with bath application (left) and local application (right) of DM. (Gray; E-cadherin and Cyan; CD44). Dotted lines indicate AER-like tissue. **h** Schematic model of the induction of AER-like tissue on the tip of ES–HLB. LDN, LDN193189; DM, Dorsomorphin; E, epithelial layer; M, mesenchyme aggregate; ES–HLB, mESCs-derived hindlimb bud. Scale bars, 100 μm

From the comparative transcriptome analysis, we also noticed that Lmx1b, a dorsal LB mesenchymal marker shows lower expression in ES–LBs (Fig. 3e, g and Supplementary Fig. 9a). Using qPCR analysis, it was confirmed that the expression of the ventral marker (*msx1, en1*) was higher than that of dorsal markers (*wnt7a, lmx1b*) in the ES–LB (Supplementary Fig. 9c). However, it was not possible to detect ectodermal Wnt7a and En1 by in situ hybridization, but only by qPCR because of low expression levels. Previous studies suggested that AER is formed by balanced BMP activity between dorsal (suppressed) and ventral (activated) sides during LB formation^26–28^. Based on these results, we hypothesized that AER is not formed in the ES–LB owing to its differentiated state biased towards the ventral side. As expected, shot-term treatment with BMP antagonists (LDN, DM) on day 7 increased expression of the dorsal ectodermal marker (*wnt7a*), and the dorsal mesenchymal marker (*Lmx1b*) in ES–LB culture (Supplementary Fig. 9c, d). Interestingly, in response to BMP inhibition, thickened epithelial tissues expressing AER markers (*fgf4, fgf8 and* CD44) appeared as small fragmented parts in the surface layer with maintaining the expression of *fgf10* (Fig. 4a–d and Supplementary Fig. 9e, f). To investigate the relationship between the position of AER formation and BMP signal antagonism, we locally suppressed BMP signaling using a local injection of BMP inhibitors in an incubator combined-microscope as previously reported^29^ (Supplementary Fig. 10). In this experiment, BMP inhibitor (DM) and DiO mixed medium was applied locally from one side into the ES–HLB. As a result, thickened epithelial tissue formed at the tip of the LB region in which AER markers *fgf8*, *fgf4,* and CD44 were expressed (Fig. 4e–h, Supplementary Fig. 11a, and Supplementary Movie 4, 5). This structure was also active for Wnt signaling (7tcf+, Supplementary Fig. 11a). Unexpectedly, this tissue did not form when another BMP inhibitor LDN was used (Supplementary Fig. 11b, c). This difference may be owing to the different effect on the induction of *wnt7a* expression of DM and LDN (Supplementary Fig. 9c). These results indicated that the ES–LB was in a suitable developmental state for the induction of AER-like tissue by controlling the ventral to dorsal positional shift. However, contrary to the expectation, the D-V pattern of the internal mesenchymal cells did not follow the positional shift even when the BMP inhibitor locally induced in ES–HLB (Fig. 4c, e). This unexpected result suggested that AER induction may be adequately elicited by BMP antagonism in only the epithelial part, as suggested in previous reports^21,28,30,31^.

### Engraftment of ES–LBs into developing mouse limbs

We finally investigated the osteogenic potential of the ES–LB and its adaptability in developing limbs using three different experiments; self-chondrogenesis, whole-embryo culture and transplantation. To test the self-chondrogenesis of the ES–LB, culture was extended to day 13. On day 13, the epithelial tissue that had continuously covered the entire aggregate on day 9, lost its continuity, and thicker epithelium partly remained on the surface of the aggregate (Supplementary Fig. 12b, c). As a control, we prepared E10.5 mouse FLBs and HLBs that were excised from the base of the limb and cultured in vitro for 4 days (Supplementary Fig. 12a). In both preparations, mesenchymal cells overflowed from the region not covered with the epithelial layer. We repeatedly observed that the overflowing mesenchymal cells differentiated into chondrocytes (Sox9^+^, Alcian Blue stain) (Supplementary Fig. 12a–d). However, expression of marker genes for cells invading into the LB from adjacent tissues, such as myoblasts, motor neurons, and angioblasts^32^ was barely detectable in ES–LBs. (Supplementary Fig. 12d). To examine whether ES–LBs could differentiate into a mature state of osteocytes, ES–LBs (*pCAG-H2B::mCherry* line) were transplanted into the capillary-rich renal capsule^33^ (Supplementary Fig. 13a). As a result, both, the mouse FLB and ES–LB matured into cells of each stage of osteogenesis^34^; immature cartilage (Alcian Blue^+^, Sox9^+^), mature cartilage (Alcian Blue^+^, Runx2^+^, Type X collagen; Col10^+^), and bone (Runx2^+^, Type 1a1 collagen; Col1a1^+^) (Supplementary Fig. 13b, c). Thus, these results indicate that the ES–LB has an ability to give rise to skeletal formation. To investigate whether ES–LBs are capable of accepting cells invading from surrounding tissues, the ES–LB was attached to the posterior region of the base of the E10.5 mouse FLB and cultured for 48 h in a whole-embryonic culture system (WEC)^35^ (Supplementary Fig. 14a, c). As a positive control, we prepared E9.5 to 10.5 mouse forelimb and HLBs, which were dissected in the posterior region. We also prepared ES-derived optic cup as a negative control. (Supplementary Fig. 14b, d). Successful engraftment was assessed by the integration of epidermis (p63^+^) between the host and graft. Mouse FLBs and ES–LBs were engrafted into the host embryo, however, HLBs had a lower graft survival rate than FLBs. In contrast, the ES–derived optic cup could not connect with the LB region. Interestingly, myoblasts (myogenin^+^) and neuronal fibers (neurofilament^+^) invaded ES–LB grafts from the host embryo with high probability, as observed in mouse LB grafts (Supplementary Fig. 14e, f and Supplementary Table 1).

**Fig. 5:**
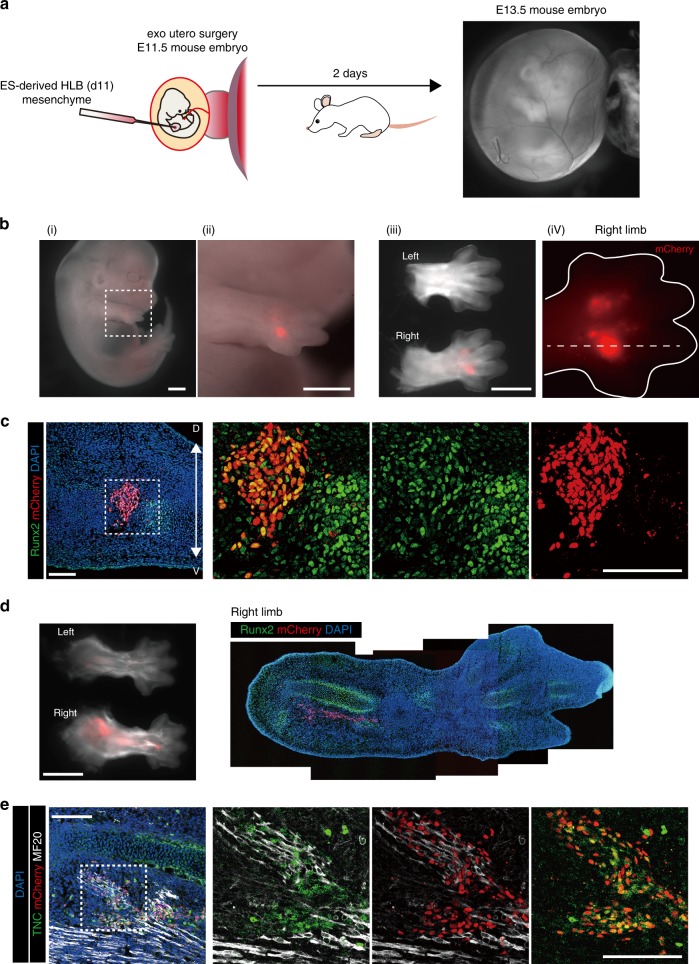
ES–HLB mesenchyme differentiates into digit osteoblast in developing limb bud. **a** Scheme of exo utero transplantation. **b** Two days post grafting. ES–HLB (pCAG-H2B::mCherry line) grafted into embryonic right-forelimb bud. ii shows an enlarged view of the dotted box of i. iii shows a comparison between non-grafted (upper) and grafted (lower) forelimb bud. iv shows enlarged view of mCherry + transplant site in the right-forelimb bud. White line and dotted vertical line indicate the limb bud form and sectional position, respectively. Scale bars, 1 mm. **c** Immunostaining of transverse section of the grafted position (**b**, iv). ES–HLB mesenchymal cells (mCherry+) were organized into osteogenesis of digit (Runx2+). **d** Mouse hindlimb bud at 2 days post transplantation. ES–HLB (pCAG-H2B::mCherry line) grafted into right hindlimb bud (**d**, left panel). The sagittal section of transplanted right-limb bud indicates ES–HLB mesenchyme (mCherry+) adapted limb development along with the limb elongation (**d**, right panel). **e** Sagittal sections showing ES–HLB mesenchyme (mCherry+) differentiated into tendon (TNC+) as part of muscle (MF20+)-tendon unit (the right pictures indicate higher magnifications of the white dotted box areas in the each of the left picture). Scale bars, 100 μm

Finally, we examined whether the ES–LB could be incorporated into developing LBs without disturbing the normal developmental process and could differentiate into multiple cell types of limb progenitors. To do this, we performed an exo utero surgery^36^ to graft ES–LBs into the embryonic LB (Fig. 5a). Isolated mesenchyme from ES–HLB (*pCAG-H2B::mCherry* line) on day 10 were injected into the presumptive autopod region of the E11.5 mouse FLB or HLB. Two days after transplantation, we collected the host embryo, and examined the state of the developmental host limb and the compatibility of grafted cells. As a result, the limbs on the side where the cells were transplanted developed morphologically normally as well as on the non-grafted limbs (Fig. 5b, d). In contrast to the IM-like cells transplantation (Supplementary Fig. 3 and Supplementary Fig. 15), grafted cells expanded in the host LB and were distributed in the prospective humerus or femur to digit-forming area (Fig. 5b, d). Despite the state of pre-chondrogenesis of the transplanted cells (Supplementary Fig. 12d), it was found that these cells adapted to the host environment and differentiated into multiple lineages including chondrocytes (Runx2-positive) and tenocytes (Tnc-positive) without interfering with the development of the host limb (Fig. 5c, e). Taken together, these results clearly indicated that ES–LBs have a multiple differentiation potential in the developing LB.

## Discussion

**Fig. 6:**
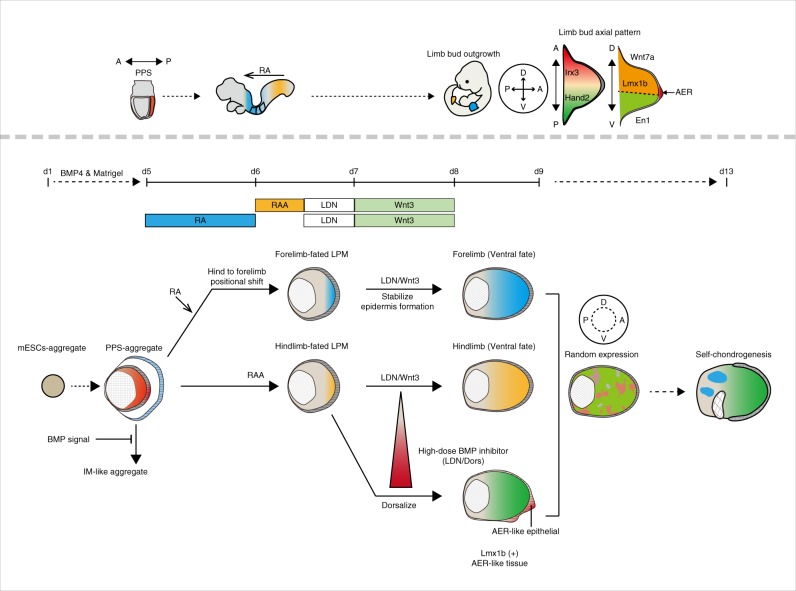
Schematic model of the differentiation of ES–LB. The developmental process of mouse embryo from E7.5 to 10.5 (above gray dotted line), which correlated with each time-course of the ES–LB differentiation. mESC aggregates differentiate into PPS aggregate by applying BMP4 and matirgel on days 1–5. PPS-aggregates demonstrate mesodermal lineage shift from LPM to IM by inhibiting BMP signal. PPS-aggregates differentiate into LPM, and regional identity of fore or hindlimb bud-derived LPM could be controlled by adjusting RA signal activity. On days 7–8, Wnt3 effectively stabilizes thickened ectoderm. On day 9, ES–LB shows limb bud-like morphology, however, the state of gene expression indicates ventral-biased and no reproducible pattern. On the other hand, the addition of high-dose LDN (1 μM) or DM (10 μM) on days 6.5–7 elicit dorsalization (Lmx1b+) and induction of AER-like tissue in the ES–LB ectoderm. On day 13, ES–LB indicates self-differentiation of immature cartilage. ES–LB, mESCs-derived limb bud; PPS, posterior primitive streak; A-P, anterior–posterior; D-V, dorsal-ventral; RA, retinoic acid; RAA, retinoic acid antagonist; LDN, LDN193189 (BMP inhibitor); DM, dorsomophin (BMP inhibitor); IM, intermediate mesoderm

In summary, here we report the formation of organoids with characteristics similar to trunk appendages consisting of mesenchymal cells covered with epidermis (Fig. 6). Induction of LB-like tissue from murine ESCs was achieved by stepwise manipulation of exogenous factors, which have been known to play key roles in regional patterning and lineage determination in vivo. Here, we showed that a complex LB-like structure is self-organized from homogenous mESC aggregates in the presence of Matrigel and BMP4 in a initial culture condition, and that both exogenous and endogenous BMP signals play crucial roles in inducing PPS, fate determination to LPM and to the formation of the surface epithelium. However, it remains unclear as to how the epithelial tissue is derived from mESCs and how it forms the epidermis-like surface structure, also the molecular mechanisms by which the LB mesenchymal cells are specified from multilineage mesodermal progenitors derived from mESCs remain to be elucidated. Although the comparative transcriptome analysis revealed that ES–LBs have a positional identity similar to developing LBs, we also noticed that there are substantial differences between in vitro and in vivo situation. The overall size of ES–LB is relatively smaller than that of LBs, and there is no rapid elongation in the distal direction as seen in developing LBs. From the analysis of RNA-seq and marker expression patterns, it is considered that the following two phenomena are involved in that. One is that ES–LB might not be supplied with sufficient nutrients necessary for the proliferation and survival of internal mesenchymal cells. In developing LBs, nutrients are supplied from the outside through the blood vessels. On the other hand, in the ES–LB, a blood vessel-like structure is not formed and the nutrient supply to the inside may be insufficient. Consistent with this, substantial proliferation of internal mesenchymal cells were observed, though cell death was also found to occur more frequently than developing normal LBs (Supplementary Fig. 5b). The cause of the distal elongation being inhibited may also be owing to the loss of expression of ectodermal *fgf8* in AER. It was reported that in a conditional knockout mouse (*Fgf8*^*c/c*^*; AP2-Cre*), which lacks expression of *fgf8* in the epidermis, LB elongation does not occur, and proliferation in mesenchymal cells decreases, whereas cell death increases as seen in ES–LBs^37^. Therefore, it is assumed that although ES–LB shows tissue morphology and gene expression resembling the early LB, the distal outgrowth and axial patterning does not occur owing to insufficient formation of AER and insufficient nutritional supply. We also noticed that ES–HLB shows higher expression of tendon and cartilage differentiation markers, suggesting that the differentiation state of the cell is more advanced than early LB (Fig. 3f, g).

In particular, levels of some patterning-associated molecules are decreased in ES–LBs owing to the lack of signals from the signaling centers (AER, ZPA). As we demonstrated in this study, the positional shift from ventralize to dorsalize led to the formation of AER-like tissues in ES–LBs, suggesting that the epidermis in ES–LBs has adequate potential to form AER in response to the anisotropic environment. However, that still has not reached an ample recapitulation of a LB organoid because of the lack of the continuous proximal–distal growth and the patterning, which are established by AER and ZPA in in vivo LB. We tried to culture for a more extended period to observe the pattern formation of inner mesenchymal differentiation after the artificially induced AER-like tissue. However, it was not possible to culture LB-like tissue derived from ES cells for more than 10 days while maintaining a complex state of epithelial and mesenchymal cells in a healthy state. It was probably because the invasion of blood vessels did not occur in the LB-like tissue derived from ES cells and could not supply nutrients internally. Thus, our method can induce LB-like tissue in the early state from ES cells but has the limitation that it cannot recapitulate later limb development such as digit patterning. To overcome this limitation, it might be necessary to form other tissues such as blood vessels and nerves inside.

Results from functional analysis demonstrated that ES–LB mesenchyme differentiated into cartilage without supplementing it with any exogenous factors^38^. Furthermore, ES–LBs showed endochondral ossification in the renal capsule. Taken together, our results demonstrated that ES–LB mesenchyme has potential for self-osteogeneis in vitro, which raises the possibility that it might be a promising approach for a chondropathy model. Furthermore, the embryo recognizes the ES–LB as a LB, and it allows integration of myoblasts and neurons, which are necessary for functional limb development. In addition, we confirmed the potential of ES–LBs to develop into a limb by using exo utero surgery. Our transplantation studies provide ample evidence for the capability of the ES–LB for contribution to developing limb without disturbing the normal development, in contrast to the situation in which ESC-derived IM-like cells were grafted. It may be possible in the near future to make a stand-alone limb from PSCs. From the perspective of regenerative medicine, it is reported that the formation of blastema is necessary for regeneration of limbs as seen in amphibians^39^. As blastemas and LBs are known to have homologous structures and gene expression patterns, LB-like tissues derived from human PSCs may be useful for regenerating damaged limbs and digits of mammals in future studies.

## Methods

### Maintenance and differentiation culture of mESCs

The maintenance medium contained G-MEM supplemented with 1% FBS (JRH), 10% knockout serum replacement (KSR; Invitrogen), 0.1 mM non essential amino acids (Gibco), 1 mM pyruvate (Gibco), 0.1 mM 2-mercaptoethanol (Nacalai), 2000 U/ml LIF, 1 µM PD0325901 (Wako), and 3 µM CHIR99021 (Stemgent). For SFEBq culture, ES cells were dissociated to single cells in 0.25% trypsin-EDTA (Gibco) and quickly re-aggregated in differentiation medium (3000 cells per 100 µl per well) in 96-well low-cell-adhesion plates (Lipidure Coat, NUNC) (Day 0). Differentiation medium was G-MEM supplemented with 1.5% KSR, 0.1 mM non essential amino acids, 1 mM pyruvate, and 0.1 mM 2-mercaptoethanol. From day 1 to 5, human BMP4 (R&D) and matrigel (growth-factor-reduced; BD Biosciences) were added to culture to final 10 ng/ml and 4% (v/v), respectively. The culture was transferred to bacterial-grade plastic petri dish on day 5, and amnion-like membrane removed by sharpened tweezers (Supplementary Movie 2 which was acquired by attached digital camera in Olympus SZX16) for maintenance healthy culture condition of inner PPS-aggregates. For hindlimb differentiation, PPS-aggregates cultivated in the 2.5% KSR differentiation medium on days 5–6. From day 6, medium changed LB-differentiation medium (LB-medium) that was high glucose dulbecco’s modified eagle medium (Sigma) supplemented with ITS + 1 (Sigma), 0.1 mM 2-mercaptoethanol, and added to 1 µM AGN193109 (retinoic acids antagonist; RAA, Toronto Research Chemicals) on days 6–6.5. For forelimb differentiation, PPS-aggregates cultivated in the 2.5% KSR differentiation medium including 1 µM all-trans retinoic acids (RA, Sigma) on days 5–6, and then, replaced into LB-medium on days 6–6.5. From day 6.5, both of ES–FLB and -HLB cultivated in the LB-medium adding 4 µg/ml hydrocortisone (hydro, Sigma) and 0.8% methylcellulose (MC, Wako)^40^. For stabilization of thickened epithelial layer, 0.1 µM LDN193189 (LDN, Sigma) was added on days 6.5–7, and 50 ng/ml Wnt3 (Abnova) was added on days 7–8. After washed out medium, LB-like tissue replaced into LB-medium (+hydro and MC) on days 8–9, and then LB-like tissue formed mature LB-like structure, which composed of *Hand2::mEGFP*-positive mesenchyme covered by epithelial layer. The concentrations of regents applied to IM-like cells differentiation culture (Supplementary Fig. 3) were follows: LDN (1 µM), DM (10 µM), SB-431542 (10 µM), SU5402 (5 µM), Cyclopamine-KAAD (5 µM), and IWR1e (10 µM). For premature cartilage differentiation, LB-like tissue and E10.5 mouse embryonic LBs were cultivated in LB-medium (+hydro, +MC) for 4 days with changing medium every second day.

### Generation of knock-in and transgenic mouse ES cell lines

The gene-targeting strategy and vector construction for *Hand2::mEGFP* is as illustrated in Supplementary Fig. 1^13,41^. CRISPR-Cas9 system was performed using the GeneArt CRISPR Nuclease Vector (Invitrogen). The guide RNA sequences were designed in exon2 of mouse *hand2* gene as follows; forward 5′-CGCCAGCCGCTGCAGCCACG-3′, reverse 5′-CGTGGCTGCAGCGGCTGGCG-3′. To generate the targeting construct, the 5′ arm (1.5kbp) and 3′ arm (1.0 kbp) were amplified by PCR from BAC plasmid (bMQ195f16). Homologous recombinant ES cells were selected with G418, and the targeted clones were confirmed by genomic PCR and sequencing (5′ primer 5′-GAGGAGCCTCTGACGACATATATTA-3’, 3′ primer 5′-ACTGTGCTTTTCAAGATCTCATTCT-3′). *7Tcf::Cherry* and *pCAG-H2B::mCherry* (the *β-catenin::mEGFP* site was replaced with the *H2B::mCherry*)^13^ vectors were individually transfected into *Hand2::mEGFP* knock-in mESCs line.

### qRT-PCR for gene expression analysis of ES–LBs and embryonic tissues

We used 8–10 of whole mESC-derived cell aggregates and 3 of amputated-mouse LB in each qRT-PCR analysis.

For FACS analysis, cells were counted with a FACSAria (Becton Dickinson), and the data were analyzed with the FACSDiva software (Becton Dickinson). *Hand2::*mEGFP-positive mesenchyme were sorted from 96 aggregates of PPS-like tissues on day 5 and LB-like tissues on day 9 in each experiments (Fig. 3, Supplementary Fig 2d, and Supplementary Fig. 7).

For analyzing the expression of AER and D-V polarity markers (*fgf4, fgf8, wnt7a*, and *en1* in Fig. 4a, b), LB-like tissues were treated with 2.5 mg/ml dispase II (Roche) in LB-medium for 1 h at 37 °C, and epithelial layer was mechanically peeled away from mesenchymal aggregate by using a sharpened tungsten needle.

Total RNA purified from samples using the RNase micro kit (QIAGEN) and cDNA synthesis was performed by using SuperScriptII (Invitrogen). qRT-PCR was performed with Power SYBR Green PCR Master Mix by the QuantStudio 6 and 7 Flex Real-Time PCR Systems (Applied Biosystems) and data were calculated by standard curve method and normalized to β-actin expression. Primer sequences were described in the Supplementary Table 2.

### RNA-seq analysis

*Hand2::*mEGFP-positive mesenchyme were sorted from 96 aggregates of ES–FLB and –HLB on day 9 by using FACSAria. Mouse embryonic LB, cardiac, branchial arch, and tail bud were amputated from three of E10.0–E10.5 embryos. Total RNA purified as described above. Sequencing was performed using the Illumina HiSeq1500 platform. Using TruSeq Stranded mRNA LT Sample Prep Kit (Illumina), we constructed library and sequencing was carried out to generate single-end 80 bp reads using a 50 cycle Hiseq Rapid SBS Kit v2-HS (Illumina). Base calling was processed with RTA 1.18.64. Fastq files were generated with bcl2fastq 1.8.4 (Illumina). The quality of the RNA-seq reads was evaluated using the FastQC 0.11.5 quality check package. Having ensured high quality of the data, sequence reads for each library were mapped independently to the mouse genome assembly mm10 and RefSeq annotation, using the spliced aligner HISAT 2.0.4 with default parameter settings. This yielded a high percentage of unique and properly paired reads, ~ 88% for all libraries. Successfully mapped reads were quantified against the annotated UCSC transcriptome for mm10 to estimate the number of fragments originating from individual genes using the Cuffdiff package 2.2.1. The count data estimated by Cuffdiff was then used as the input to bioconductor package edgeR 3.16.2 to assess the biological variability in the samples and test for differential expression. mESCs publically data sets^4^ were download from NCBI GEO database (accession number GSE89270)^42^.

### Immunohistochemistry and in situ hybridization

For tissue frozen section, ES-derived tissues and LB tissues are fixed with 4% paraformaldehyde (PFA) in PBS at 4 °C, overnight. Samples were then washed in PBS, immersed in 15 and 20% sucrose in PBS at 4 °C, and frozen in OCT embedding compound. Sections were cut (10 µm) and permeabilized with 0.3% TritinX100 in PBS for 15 min, and then blocked for 15 min at RT in Blocking-One (Nacalai). For first antibody incubation, antibodies were diluted in Blocking-One and incubated overnight at 4 °C. Antibodies against the following proteins were used at the indicated dilutions: GFP (rabbit, 1:1000; MBL; rat, 1:1000; Nacalai), DsRed (rabbit, 1:500; Clontech), Oct3/4 (mouse, 1:1000; BD pharmigen), Nanog (rabbit, 1:1000; ReproCELL), Pitx1 (guinea pig, 1:3000; Custom made), Irx3 (gunea pig, 1:500; Custom made), Tbx5 (guinea pig, 1:3000; Custom made), Hand2 (goat, 1:500; Santa Cruz), PDGFRα (rabbit, 1:1000; Santa Cruz), Msx1/2 (mouse, 1:500; Santa Cruz), Meis1/2 (goat, 1:500; Santa Cruz), AP2α (rabbit, 1:1000; Santa Cruz), Brachyury (goat, 1:500; Santa Cruz), phosphor-smad1/5 (rabbit, 1:1000; Cell Signaling), FoxA2 (rabbit, 1:1000; Cell Signaling), Sox1 (rabbit, 1:1000; Cell Signaling), E-cadherin (rat, 1:1000; TaKaRa and goat, 1:1000; R&D), Sox9 (mouse, 1:1000; abcam), Isl1 (mouse, 1:500; DSHB), Evx1 (mouse, 1:100; DSHB), p63 (mouse, 1:500; Santa Cruz), CD44 (rat, 1:500; BD), Snail (mouse, 1:500; Cell signaling), Myogenin (mouse, 1:500; DSHB), MF20 (mouse, 1:1000; DSHB), Neurofilament 160 kDa (mouse, 1:1000; Millipore), Sox17 (goat, 1:500; R&D), Laminin α1/β1 (rat, 1:500; Chemicon), N-cadherin (mouse, 1:500; BD), Pbx 1/2/3/4 (mouse, 1:500; Santa Cruz), Ki67 (mouse, 1:500; BD), Cleaced Caspase-3 (rabbit, 1:500; Cell Signaling), Keratin5 (rabbit, 1:500; BioLegend), Keratin8/18 / TROMA-1 (rat, 1:1000; DSHB), Runx2 (rabbit, 1:500; abcam), Collagen X (mouse, 1:500; abcam), Collagen 1a1 (rabbit, 1:500; GeneTex). The antiserum against Lmx1b was raised in guinea pigs (IBL Co., Ltd., Japan) against a synthetic peptide based on Jessell, T.M. laboratory resources (http://sklad.cumc.columbia.edu/jessell/index.php). Counter nuclear staining was performed with 4,6-diamido-2-phenylindole (DAPI; Nacalai). Stained sections were analyzed with an LSM710 confocal microscope (Zeiss). For whole mount immunofluorescence, tissues were fixed with 4% PFA in PBS at 4 °C, overnight. Samples were then washed in PBS, permeabilized 1% triton in PBS at RT 1 h, and then blocked for 2 h at RT in Blocking-One (Nacalai). For first antibody incubation, antibodies were diluted in Blocking-One and incubated overnight at 4 °C, washed 0.05% tween-20 in PBS. Second antibodies were diluted 0.05% tween-20 in PBS and incubated 6 h at 4 °C. Stained samples were analyzed with Lgihtsheet Z.1 (Zeiss). 3D reconstruction of Lightsheet-captured images was visualized with Imaris software (Bitplane). For section in situ hybridization, we prepared antisense mRNA probe of *Fgf4, 8, 10*^43^. For Alcian Blue staining, tissue section samples were stained by 0.05% Alcian Blue (Sigma) staining solution in 75% ethanol: 0.1 M HCl (4:1) at 37 °C, overnight^44^. The von Kossa staining was performed by using Calcium stain Kit (Modified Von Kossa, ScyTek Laboratory, Inc.). Alcian Blue and von Kossa samples were co-stained with Nuclear Fast Red (Vector Laboratotries). Image of in situ and Alcian Blue- or von Kossa-stained sections were captured with DMi8 microscope (Leica).

### Local application system for LB-like tissue

Photolithography process was employed to fabricate the poly-dimethylsiloxane (PDMS; Dow Corning, MI, U.S.A.) mold for agarose wall. First, a silicon substrate was soaked with a mixture of hydrogen peroxide and sulfuric acid (1:2, v/v) for 10 min to remove particles and residual chemicals on the substrate. Then, the substrate was rinsed with deionized water for 5 min twice, followed by dehydrate process in an oven at 140 °C for 20 min. A 500 mm-thick negative dry film photoresist (SUEX D500; DJ MicroLaminates Inc., MA) was laminated onto the silicon substrate. Ultraviolet light irradiation was then conducted over a photomask with the desired geometric pattern, followed by a developing process involving 2-methoxy-1-methylethyl acetate. Once the photomask pattern was transferred to the photoresist coating, the silicon substrate was used as a mold for producing a PDMS mold incised with the pattern. PDMS was poured onto the silicon mold and incubated in an oven for 20 min at 90 °C after degassing for 10 min. Once the PDMS was cured, it was detached from the silicon mold to complete PDMS mold. Then, PDMS mold was placed on a glass bottom dish and preheated 6% agarose gel was poured under PDMS mold. After gelatinization, PDMS mold was removed the designed pattern on PDMS was transferred to agarose. In the local application system^29^, injection pressure was regulated by compensation pressure (Pc: 10–15, FemtoJet 4i; eppendorf). Before the local injection, 0.1 µM LDN was bath-applied for 3 h and then ES–HLB was placed between agarose gel wall. Labeling mix medium [DiO (1:500, molecular probes)-diluted LB-medium (+hydro and MC) with Dextran (Alexa Fluor^TM^ 647; 5 µg/ml, molecular probes)] supplemented with Dorsomorphin (DM; 10 µM, TOCRIS) or LDN (1 µM)] was locally injected at arbitrary side point of ES–HLB in LB-medium (+hydro, MC, and 0.1 µM LDN) for 4 h. After the local injection, ES–HLB proceeded culture in the same method of ES–LBs culture as described above until day 9. Fixed sample was mount in OCT which positioning respect to DiO labeling region located on a side as indicator of injected point. Quantitative analysis of thickness (E-cadherin positive DAPI luminance) and CD44-positive region of ES–HLB was analyzed by using ImageJ. LB region was linearized by using Polar Transformer (Plugins), and epithelial layer region extracted based on E-cadherin or p63-positive region. After corrected image value for 500 pixel, in a selected-epithelial region, DAPI and CD44 luminance value were calculated.

### Transplantation

All animal procedures comply with all relevant ethical regulations and guideline for animal studies, were approved by Research Ethical Committee, RIKEN and Kyoto University and licensed by the Japan Ministry of Education, Culture, Sports, Science and Technology and the Science Council of Japan. Mice were obtained from Japan SLC, Inc.

In LB-like tissue transplantation with WEC system, we prepared pregnant female mice and dissected E9.5-10.5 ICR or C57BL/6-Tg (*CAG-EGFP*) mouse embryo in the HBSS (Gibco) at room temperature. As a positive control of graft, we excised the posterior half of fore or HLBs from E9.5 and E10.5 mouse embryo. Hnad2-GFP-positive LB region was mechanically excised from FLB- or ES–HLB on day 9. As a control of another mESC-derived tissue, we prepared Rx-GFP^+^ neural epithelial which was mechanically excised from mESCs-derived optic cup on day 7. Each transplant tissue was holed by sharpened tungsten needle (φ 0.01 mm) at a slit region of host FLB posterior part, and cultivated in the WEC-medium [100% Rat serum (Charles river) supplemented with glucose (2 mg/ml, Wako) and penicillin/streptomycin (1:400, Gibco)] for 48 h in the WEC using the rotator-type bottle system (Ikemoto Scientific Technology)^35^.

In subcapsular transplantation of tissue in the kidney^33^, we prepared 8-week-old male ICR mice as recipients. Mice were anaesthetized with intraperitoneal injection of pentobarbital (40 mg/kg). We implanted each 2–4 of mouse FLBs on E10.5 (ICR) and LB-like tissues (*Hand2::mEGFP, pCAG-H2B::mCherry*) on days 9–10 under the renal capsule. At 2 weeks post transplantation, transplants were collected and were processed for section staining.

In exo utero transplantation^36^, we prepared pregnant female mice (E11.5 ICR mice) as recipients, which were anaesthetized with intraperitoneal injection of pentobarbital (40 mg/kg). We prepared ES–HLB and IM-like cell aggregates (*Hand2::mEGFP, pCAG-H2B::mCherry*) on day 9–10, and peeled away epithelial layer by treatment of dispase II as described above, and collected LB-like tissue mesenchyme. Mesenchymal aggregates were dissociated with collagenase D (1 mg/ml, Roche) in D-PBS (Mg^2+^ and Ca^2+^ free, Nacalai) at 37 °C, 5% CO_2_ for 30 min^45^. After the addition of an equal volume of 10% FBS-containing HBSS (Gibco) to stop the reaction, the digest cells were pipetted and filtered using a cell strainer (35 µm nylon mesh cell strainer, BD). Cells were transferred into 1.5-ml tube, and centrifuged and the supernatant discarded. The pellet was suspended in 5 µl of ice-cold 10% FBS HBSS with 0.01% Nile Blue (Sigma). In total, 1–2 × 10^5^ cells were injected into E11.5 mouse LB using glass capillary. After 2 days post transplantation, grafted and non-grafted LBs were collected and were processed for section staining.

### Reporting summary

Further information on research design is available in the Nature Research Reporting Summary linked to this article.

## Supplementary information

Supplementary information_new

Movie1

Movie2

Movie3

Movie4

Movie5

Description of Additional Supplementary Files

Reporting Summary

## Data Availability

The authors declare that all data supporting the findings of this study are available within the article and its supplementary information files or from the corresponding author upon reasonable request. Raw data for the transcriptome analysis have been deposited in the GEO database under accession code: GSE126387.
