# Supplementary material for: Self-organized formation of developing appendages from murine pluripotent stem cells
Source: Nat Commun. 2019 Aug 23;10:3802. doi: 10.1038/s41467-019-11702-y (PMC6707191; doi:10.1038/s41467-019-11702-y)
Supplement: Supplementary file 1 — Supplementary information_new [file 41467_2019_11702_MOESM1_ESM.pdf]

Supplementary information

Self-organized formation of developing appendages from pluripotent stem cells

Mori et al.

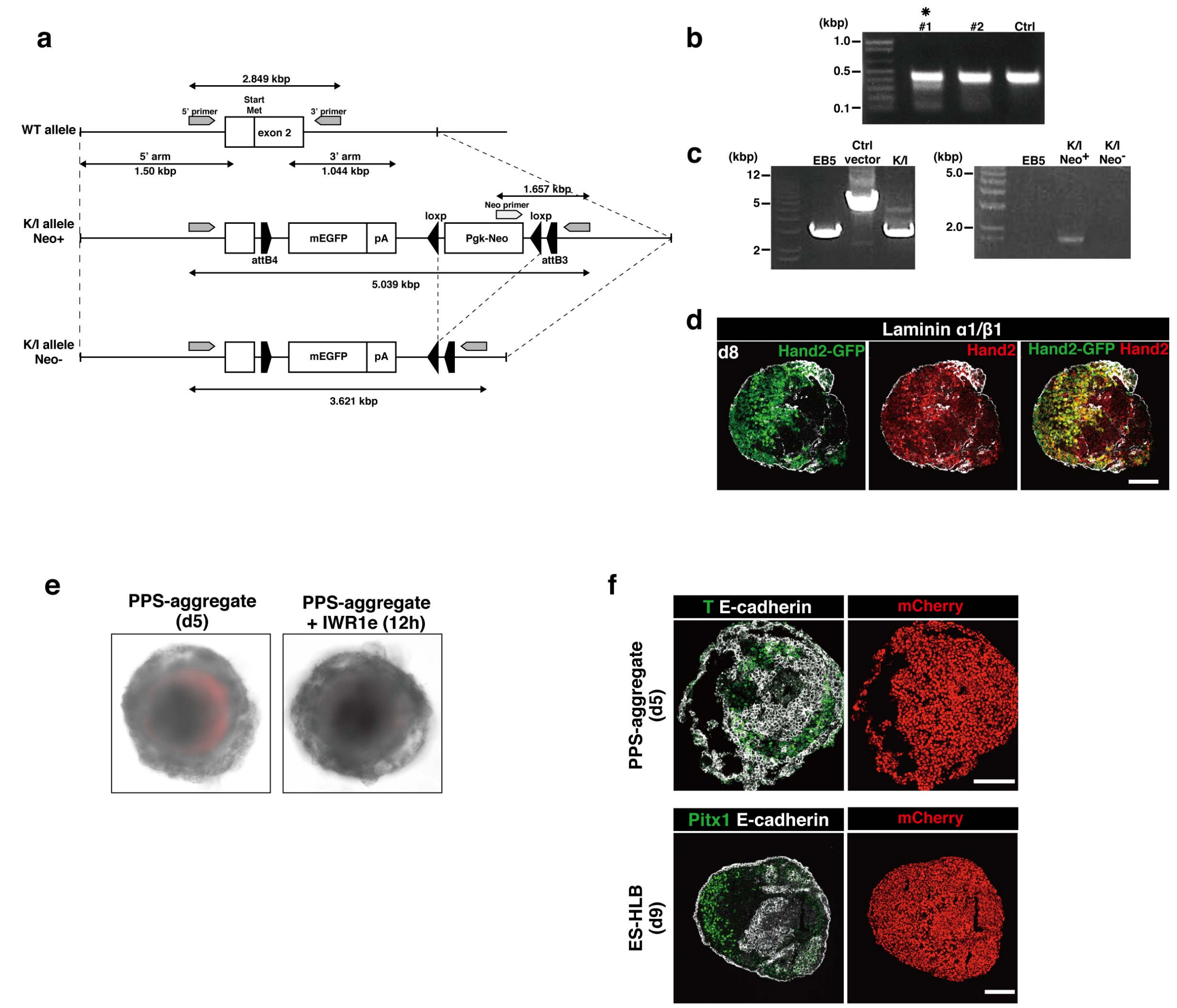

Supplementary Figure 1 mESCs reporter lines (a) Schematic model of *Hand2::mEGFP* knock-in locus. (b) The cleavage efficiency of guide-RNA. #1 target site of guide-RNA shows high cleavage efficiency (asterisk). (c) PCR genotyping of the *Hand2::mEGFP* K/I allele by using 5' and 3' primers. EB5 ES cell lines and control vector are used for the positive and negative control, respectively (left). The neomycin resistant cassette (Pgk-neo) is checked using Neo and 3' primers (right). (d) Immunostaining of ES-HLB on day 8. Endogenous Hand2 expression is co-localized with Hnad2-GFP. The basal membrane of the epithelial layer is indicated by Laminin α1β1 staining. (e) *7Tcf::Cherry* transgenic mESC-derived PPS-aggregate indicates the activation of 7Tcf on day 5 (left). Wnt inhibitor IWR1e (10 μM) effectively decreased 7Tcf activity (right). (f) Cryosection of *pCAG-H2B::mCherry* transgenic mESC line-derived PPS-aggregate on day 5 (top, T+ cells observed in inner cell aggregate) and ES-HLB on day 9 (bottom, Pitx1+ mesenchyme are covered by E-cadherin+ epithelial layer). Scale bar, 100 μm.

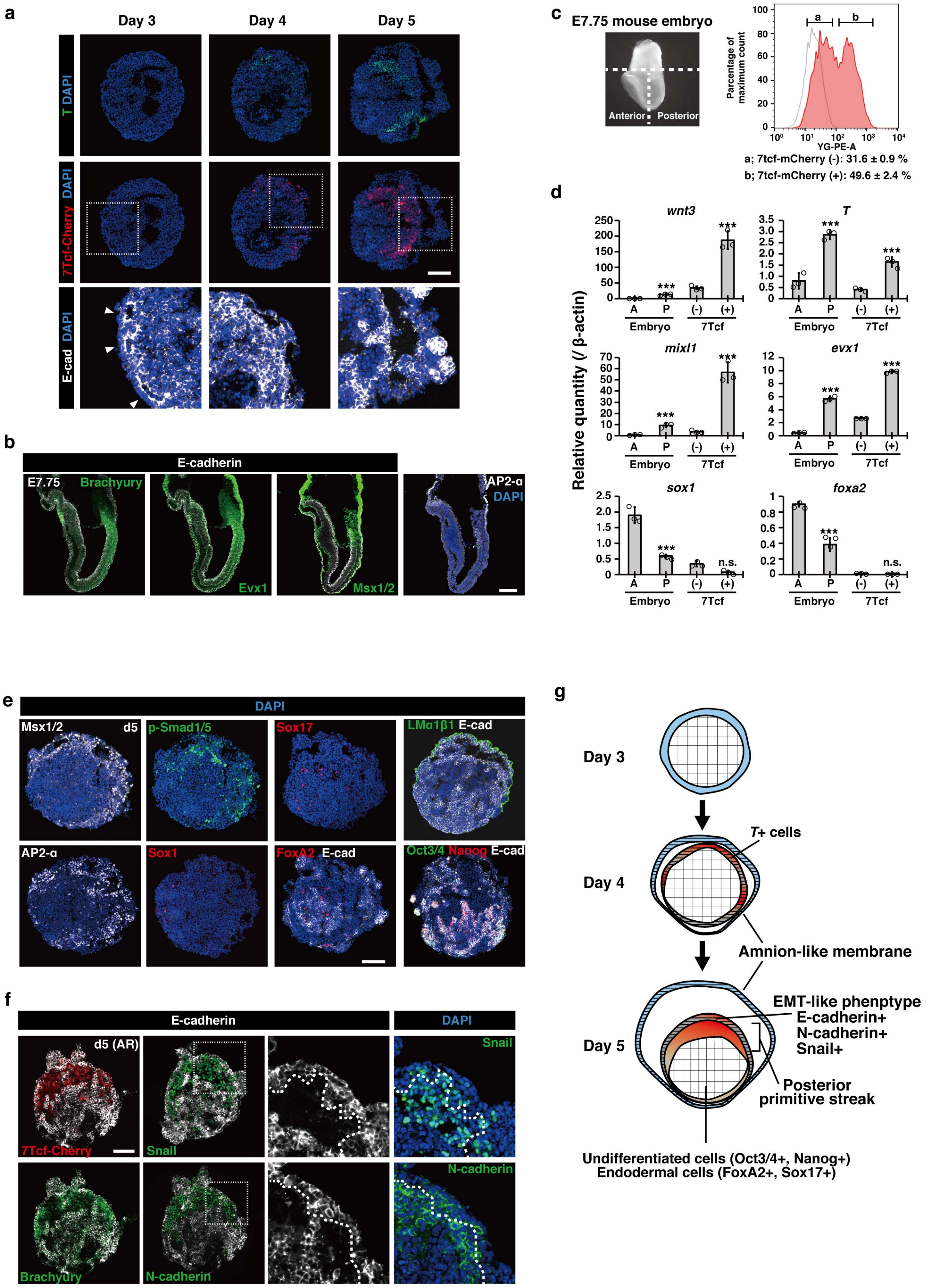

Supplementary Figure 2 mESCs aggregates differentiate into the posterior primitive streak. (a) Immunostaining of mESC aggregate on day 3 to 5 (top, T and DAPI; middle, 7Tcf-Cherry, and DAPI; bottom, E-cad, and DAPI). Bottom pictures show an enlarged

view of the dotted box area of middle pictures. Arrowheads indicate the start point of separation between the amniotic membrane and inner cell aggregate. (b) Sagittal section of the E7.75 mouse embryo. The expression of T and Evx1 indicate PPS area. Msx1/2 and AP2-α are expressed in both amnion and embryonic posterior area. (c, d) Gene expression analysis of E7.75 mouse embryo and PPS-aggregates on day 5. Bright-field view of E7.75 mouse embryo (c left, dotted lines indicate dissection area of the anterior and posterior side of the mouse embryo). FACS analysis of *7Tcf::Cherry*+ population of PPS-aggregate on day 5 (red). Grey, control undifferentiated mESCs (c, right). qRT-PCR analysis of the E7.75 mouse embryos (anterior and posterior side, mean ± s.d, n=3 independent experiments, ***P < 0.005 compared to anterior; two-tailed Student's t-test) and FACS-sorted *7Tcf::Cherry* negative (-) and positive (+) cells on day 5 (mean ± s.d, n=3 independent experiments, ***P < 0.005 compared to 7Tcf (-); two-tailed Student's t-test) (d) . (e) Cryosection of mESC-derived PPS aggregate on day 5. Msx1/2, p-Smad1/5, and AP2-α are expressed in the amniotic membrane. Neural marker (Sox1) and endodermal markers (FoxA2 and Sox17) are slightly expressed in an undifferentiated area. LMα1/β1 (Laminin α1/β1) is expressed in the basal side of the amniotic membrane. Oct3/4 and Nanog are expressed in inner cell aggregate and are partly expressed in the amniotic membrane. (f) Immunostaining of amniotic membrane removed PPS-aggregate on day 5. EMT marker genes (N-cadherin and Snail) are expressed in the E-cadherin+ epithelial layer of 7Tcf-activated region on day 5. White dotted lines indicate a basal line of the epithelial layer. (g) Schematic illustrating of the differentiation process of mESC aggregate from day 3 to 5. A, anterior; P, posterior; 7Tcf (+), *7Tcf::Cherry* positive; 7Tcf (-), *7Tcf::Cherry* negative; AR, amniotic membrane-removed; E-cad, E-cadherin. Scale bar, 100 μm.

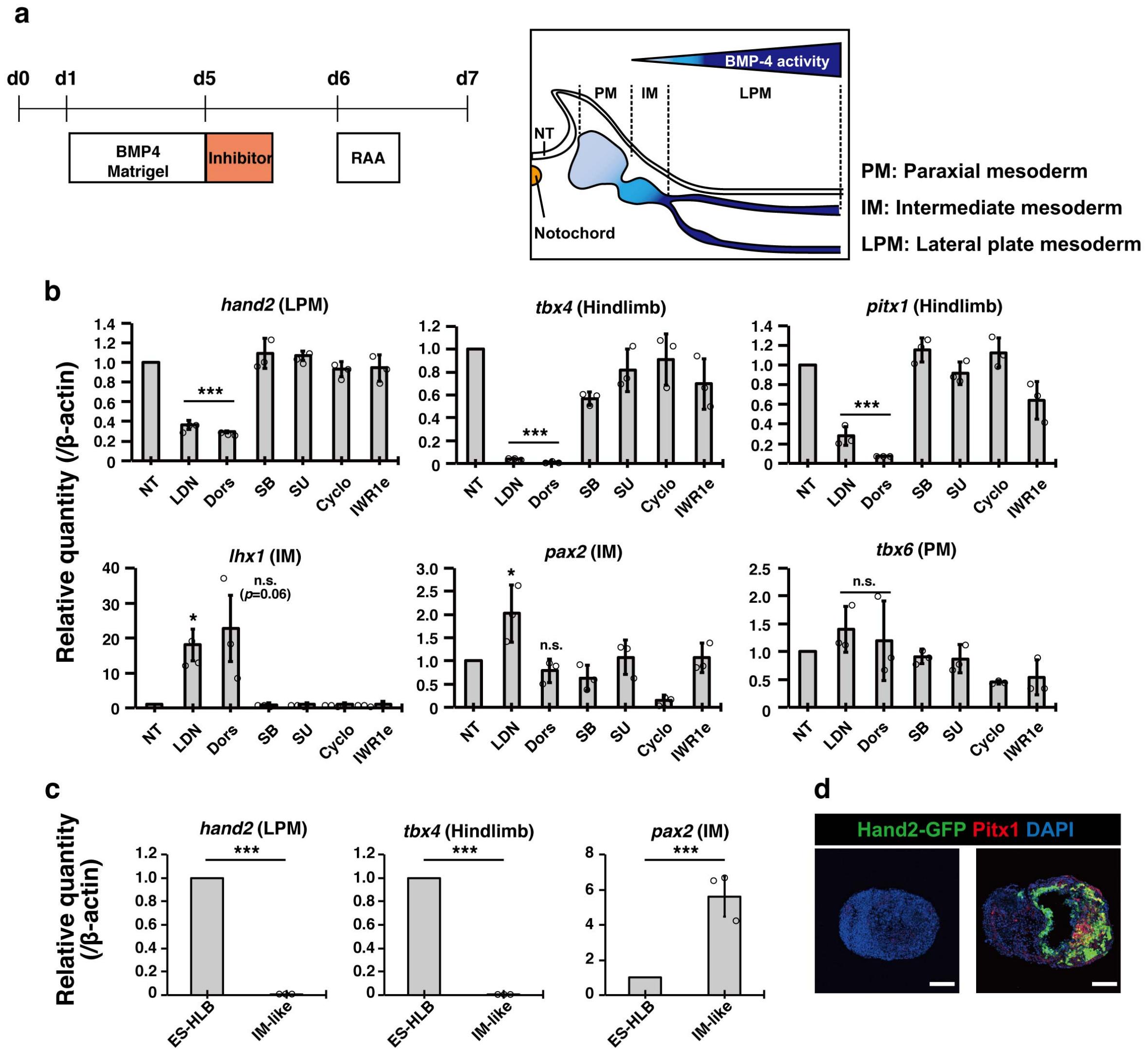

Supplementary Figure 3

BMP activity is required for LPM differentiation from PPS-aggregates. (a) Experimental timeline. Each inhibitor was added during day 5 to 5.5 (left). The right schematic model shows the relationship between the mesodermal positional value and BMP activity along with midline to the lateral side of embryo7. (b) qRT-PCR analysis of each mesodermal gene expression on day 7 aggregate. BMP inhibitors specifically changed the fate of differentiation from LPM to IM (mean ± s.d, *n*=3 independent experiments, **P* < 0.05, ****P* < 0.005 compared to NT; two-tailed Student's *t*-test). (c) qRT-PCR analysis of the gene expression on day 9 ES-HLB and IM-like cells (mean ± s.d, *n*=3 independent experiments, ****P* < 0.005 compared to ES-HLB; two-tailed Student's *t*-test). (d) Cryosection of mESC-derived IM-like cell aggregate (left) and ES-HLB (right) on day 9. Expression of Hand2-GFP and Pitx1 are clearly decreased in IM-like cell aggregates. NT, non-treat; LDN, LDN193189; DM, Dorsomorphin; SB, SB-431542; SU, SU5402; Cyclo, Cyclopamine-KAAD.

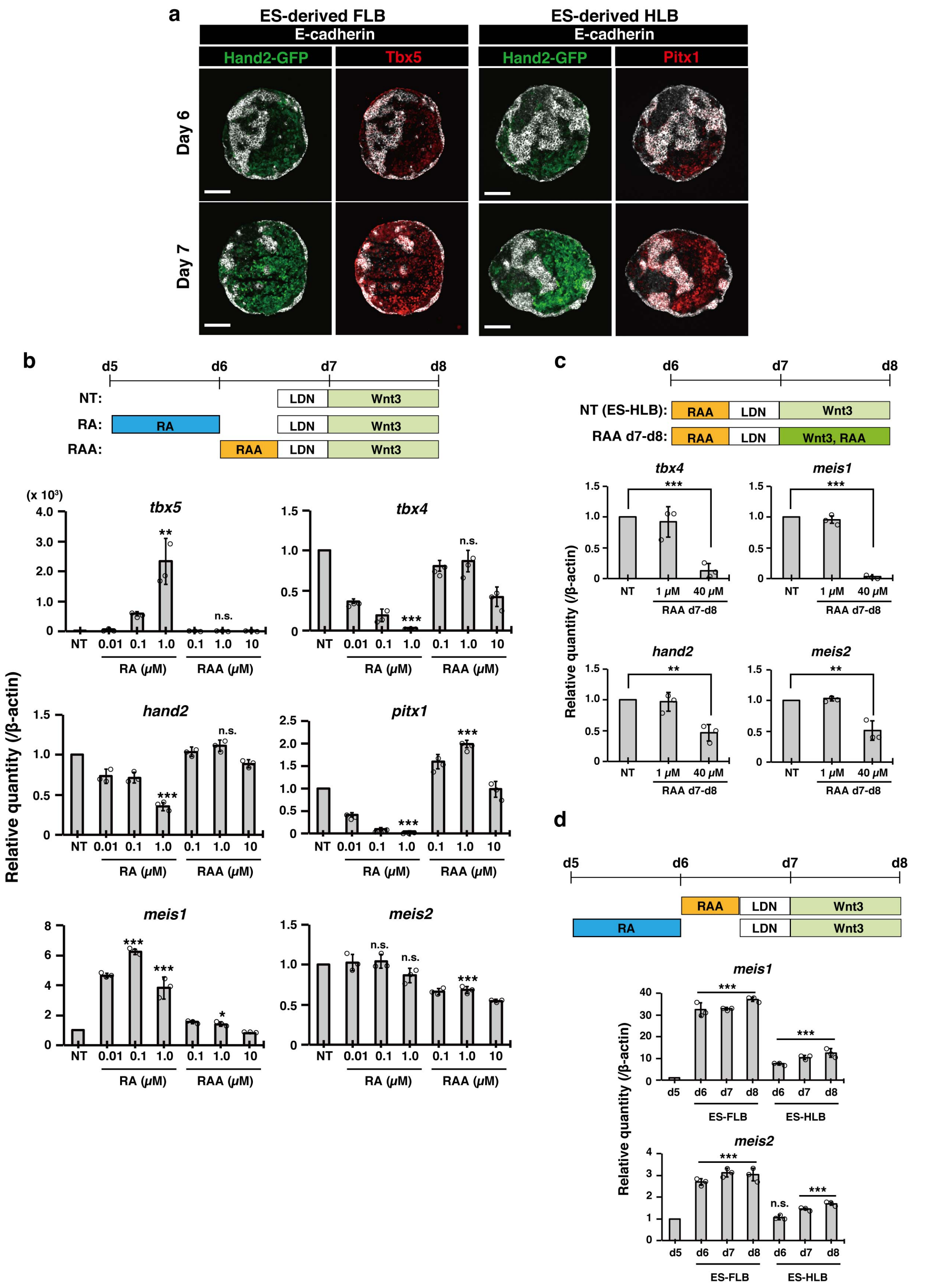

Supplementary Figure 4 RA signal regulates selective differentiation of the ES-FLB or -HLB. (a) Cryosection of ES-FLB (left) and HLB (right) on day 6 and 7. Hand2-GFP+ mesenchyme appears from day 6 which covered by ectoderm(E-cadherin+). Each of

ES-FLB and HLB is expressed Tbx5 (forelimb marker) and Pitx1 (hindlimb marker), respectively. Scale bar, 100 μm. (b-d) Experimental timelines showing the treatment period of RA and RAA (each top). (b) qRT-PCR analysis of the dose-dependent effect of the selective differentiation of ES-FLB and -HLB by adding RA (added during day 5 to 6) and RAA (added during day 6 to 6.5) (mean ± s.d, n=3 independent experiments, **$P < 0.01$, ***$P < 0.005$ compared to NT; two-tailed Student's t-test). (c) qRT-PCR analysis of the high-dose RAA effect on HLB marker expression. RAA was added from day 7 to 8. (mean ± s.d, n=3 independent experiments, **$P < 0.01$, ***$P < 0.005$ compared to NT; two-tailed Student's t-test). (d) Time course qRT-PCR analysis of meis1/2 expression of ES-FLB and -HLB from day 5 to 8. Day 5-aggregate was used as control. (mean ± s.d, n=3 independent experiments, ***$P < 0.005$ compared to NT; two-tailed Student's t-test). RA, retinoic acids; RAA, Retinoic acids antagonist (AGN193109); LDN, LDN193189.

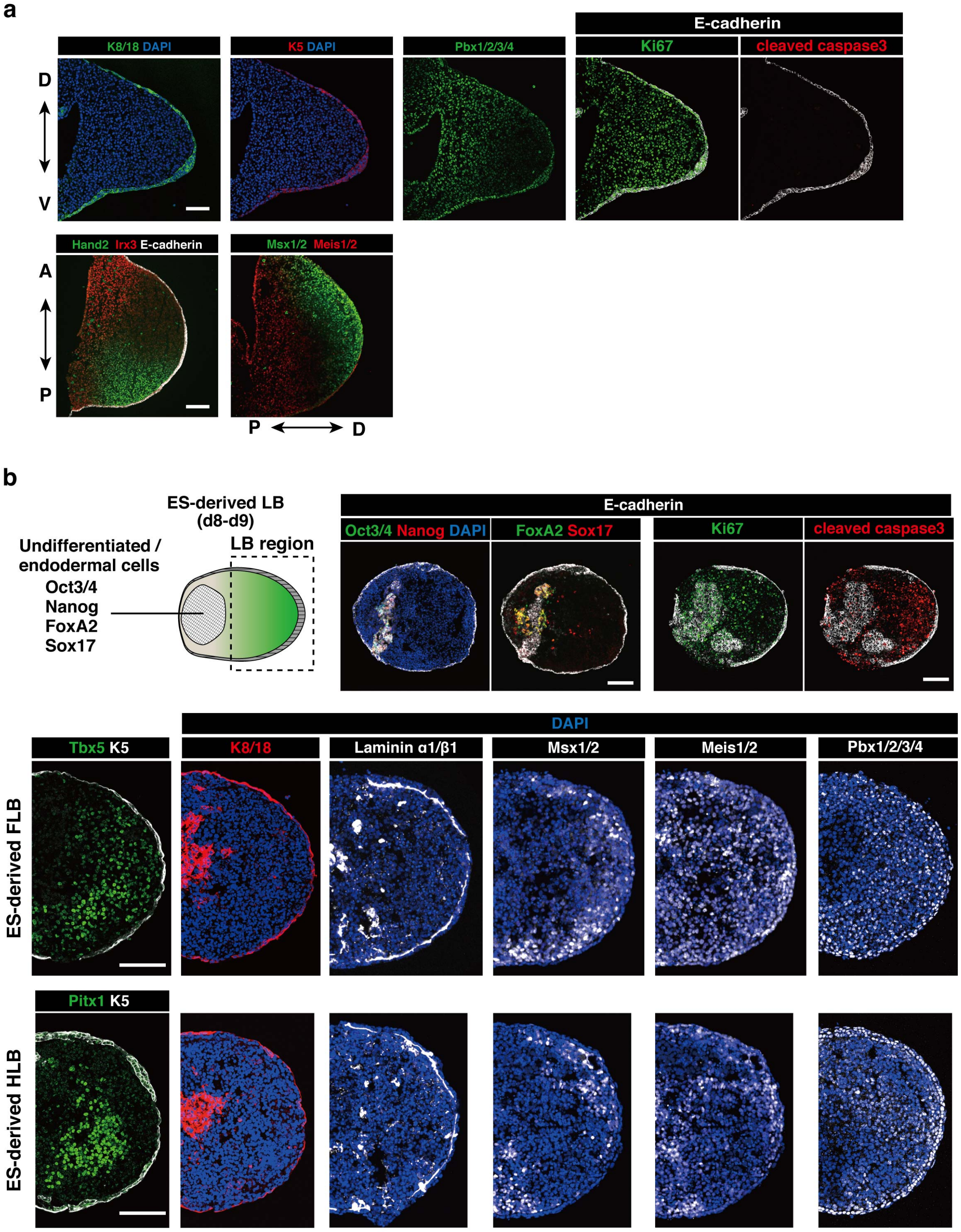
a
E-cadherin
K8/18 DAPI
K5 DAPI
Pbx1/2/3/4
Ki67
cleaved caspase3
D
V
Hand2 Irx3 E-cadherin
Msx1/2 Meis1/2
A
P
P
D
b
ES-derived LB
(d8-d9)
LB region
Undifferentiated /
endodermal cells
Oct3/4
Nanog
FoxA2
Sox17
E-cadherin
Oct3/4 Nanog DAPI
FoxA2 Sox17
Ki67
cleaved caspase3
DAPI
Tbx5 K5
K8/18
Laminin α1/β1
Msx1/2
Meis1/2
Pbx1/2/3/4
ES-derived FLB
Pitx1 K5
ES-derived HLB

c

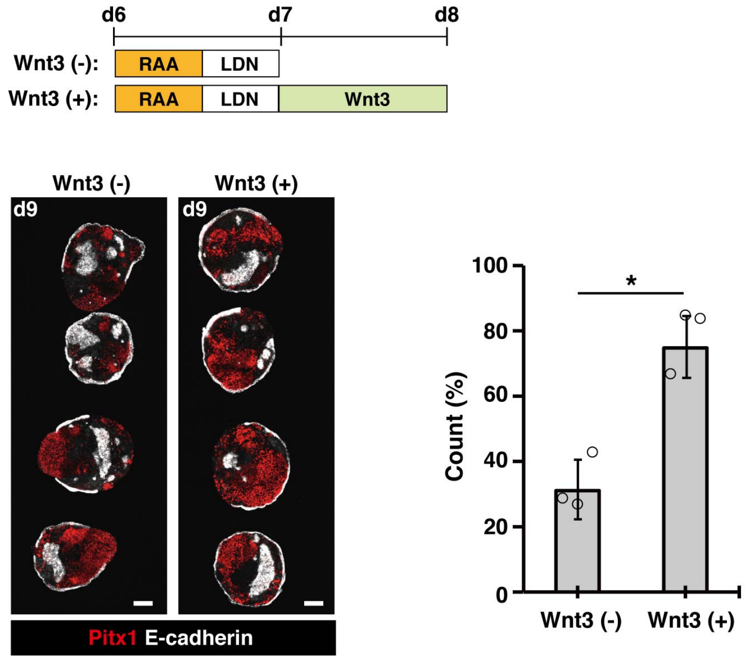

Supplementary Figure 5 ES-LB expresses limb bud molecular markers. (a) Immunostaining of E10.5 mouse hindlimb bud. Transverse sections indicate epidermis markers keratinocyte (K8/18, K5) express in limb ectoderm, early limb bud marker Pbx1-4 expresses in proximal mesenchyme and ectodermal region, and cell proliferation (Ki67) and apoptotic (cleaved caspase 3) state in limb bud (top panels). Sagittal sections indicate A-P pattern (Irx3-Hand2) and P-D pattern (Meis1/2-Msx1/2) of the limb bud. Scale bars, 100 μm. (b) Schematic of the ES-LB (top left). The opposite side of LB-region remains undifferentiated (Oct3/4 and Nanog) or endodermal (FoxA2 and Sox17) cell aggregate (top middle) Both of proliferative (Ki67) and apoptotic (cleaved caspase 3) cells exist in the LB-region (top right). Cryosection of the LB-region of ES-FLB (Tbx5) and -HLB (Pitx1) on day 9 (bottom panels). The surface epithelial layer is expressed epidermis markers (Keratinocytes, K5, and K8/18), and these basal side stained with Laminin α1/β1. Distal marker Msx1/2 is relatively expressed at distal of the LB-region, and proximal marker Meis1/2 is broadly expressed in LB-region. Early limb bud marker (Pbx1/2/3/4) is broadly expressed in LB-region. Scale bars, 100 μm. (c) Stabilization of thickened epithelial layer is increased by adding Wnt3. Experimental timeline showing ES-LBs were treated with (+) or without (-) Wnt3 (top). Cryosection of Wnt3 (-) and Wnt3 (+) ES-HLB on day 9. Mesenchymal cells (Pitx1+) partly protrude from the ectodermal pocket in Wnt (-) aggregate (bottom left). The bottom right graph shows quantitative analysis of the number of epithelial layer surrounding aggregates (means ± s.d, n=3 independent experiments every 10 aggregates, $^*P < 0.05$ compared to Wnt3 (-); two-tailed Student's t-test). A-P, anterior-posterior; P-D, proximal-distal; D-V, Dorsal-Ventral.

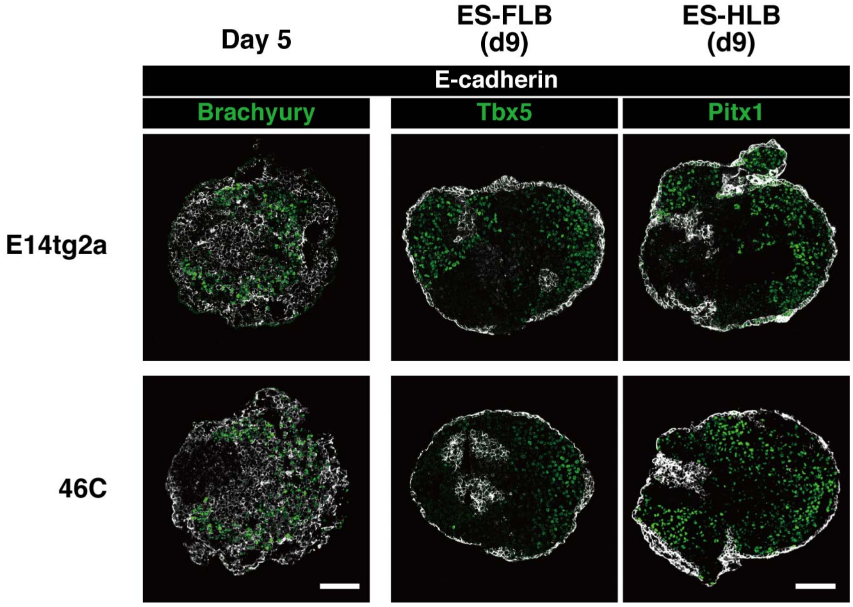

Supplementary Figure 6

Limb bud-like tissue formation from E14tg2a and 46C mESC lines. Cryosection of E14tg2a and 46C mESC line-derived PPS-aggregate and LB. Each cell lines differentiate into PPS-aggregate (Brachyury$^+$, day 5) and ES-FLB or -HLB (Tbx5$^+$ or Pitx1$^+$ mesenchyme are covered by ectoderm). Scale bars, 100 μm.

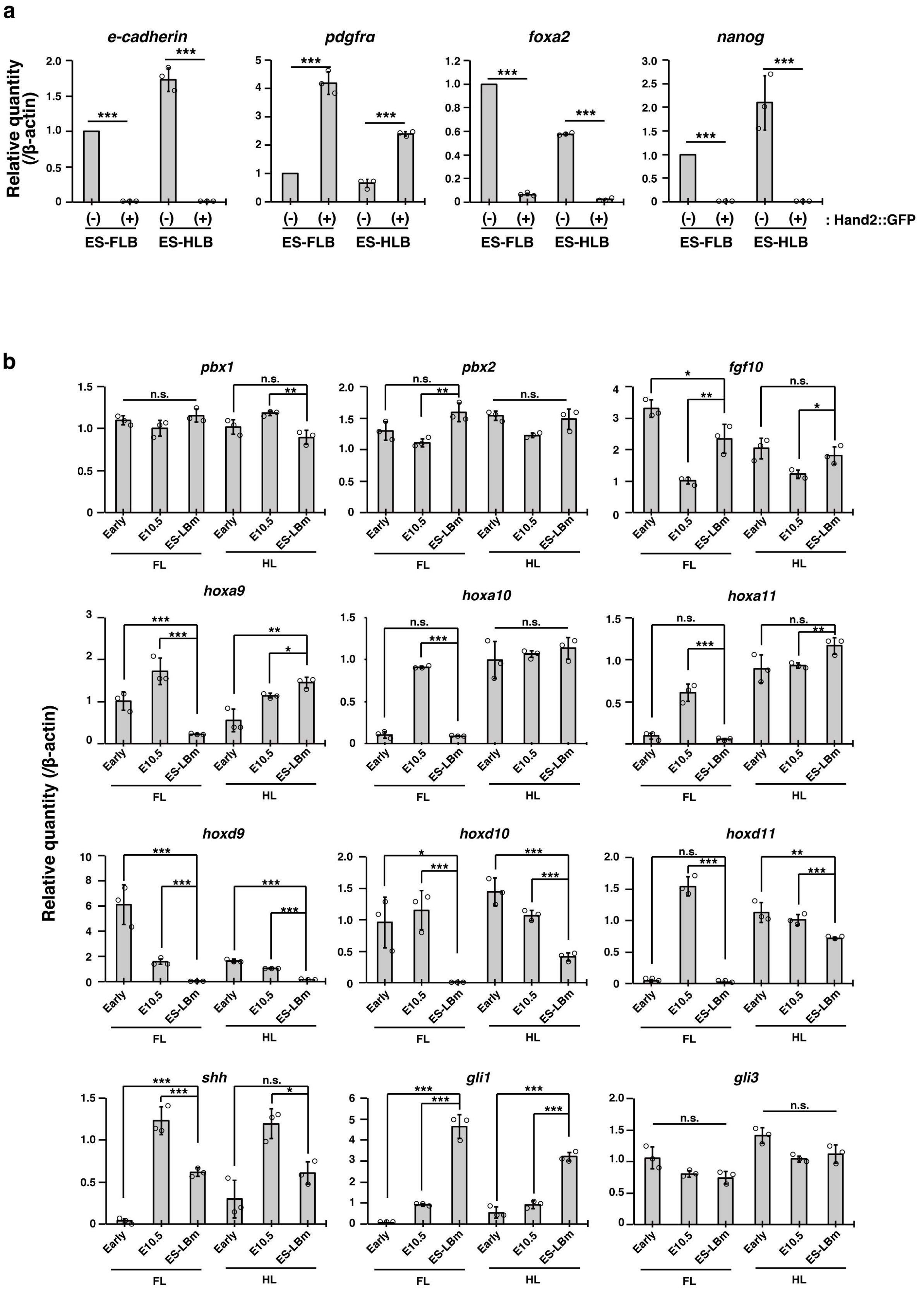

Supplementary Figure 7 Gene expression analysis of FACS-sorted *Hand2::mEGFP*⁺ mesenchyme from ES-LB. (**a**) qRT-PCR analysis shows *Hand2::mEGFP*⁺ mesenchyme effectively isolated from epithelial, endodermal, and undifferentiated cells (mean ± s.d,

n=3 independent experiments, ***$P < 0.005$ compared to ES-FLB (-); two-tailed Student's *t*-test). (**b**) qRT-PCR analysis of the limb bud mesenchyme molecular markers. *Hand2::mEGFP*$^+$ mesenchymal gene expression is compared with mouse limb buds (mean ± s.d, *n*=3 independent experiments, *$P < 0.05$, **$P < 0.01$, ***$P < 0.005$ compared to early or E10.5 limb bud; two-tailed Student's *t*-test). Early forelimb, E9.75; early hindlimb, E10.25; FL and HL, mouse forelimb bud and hindlimb bud.

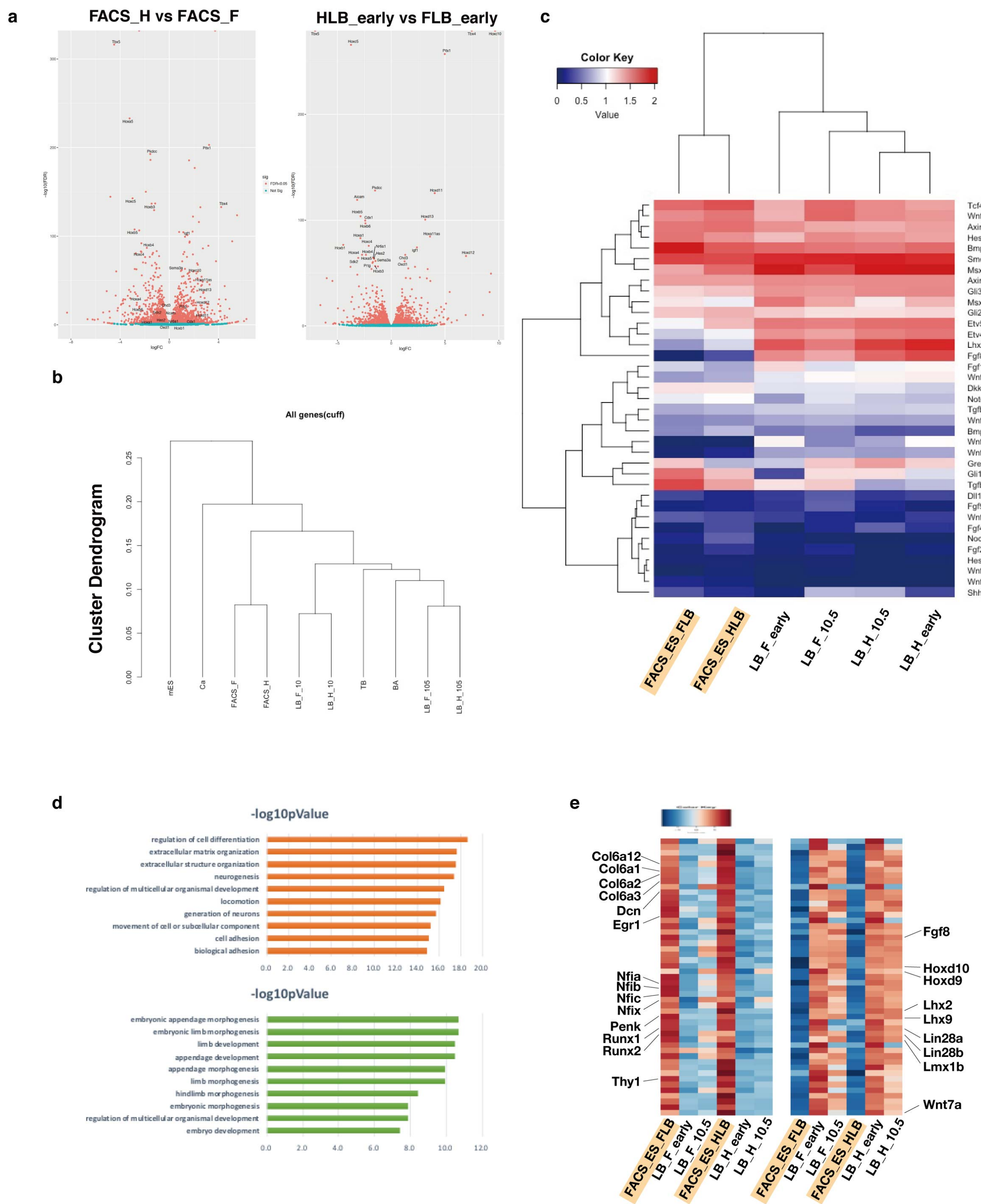

Supplementary Figure 8 Transcriptional analysis of ES-LB and mouse embryonic tissues by RNA-Seq. (a) Volcano plot for differential expression genes between ES-FLB/ES-HLB and FLB_early/HLB_early. (b) Hierarchical clustering analysis in whole transcriptome data from ES-LB mesenchyme and mouse embryonic tissues. (c) Comparative analysis of signaling molecules involved in LB development. (d) GO enrichment analysis using the top 150 genes list which is extracted by DGE analysis of the comparison between FACS_ES-HLB and LB_H_early. The upper and lower graph

shows top terms of signaling pathway which are upregulated in LB_H_early and FACS_ES-HLB, respectively. (e) Heat map visualizing the top 50 genes which are upregulated (left) and downregulated (right) in ES-HLB. Genes are extracted by DGE analysis of the comparison between LB_H_early and FACS_ES-HLB. Red asterisk, connective tissue development-related gene; blue asterisk, limb bud pattering-related gene; (e), limb bud ectodermal gene; (V), vasculogenesis-related gene; mESC, mouse ES cells; Ca, cardiac; TB, tail bud; BA, branchial arch; LB_F and LB_H, mouse fore and hindlimb bud (F_early, E9.75; H_early, E10.25; 10.5, E10.5); FACS_ES-FLB and _ES-HLB, FACS-sorted *Hand2::mEGFP*+ mesenchyme form ES-FLB and -HLB.

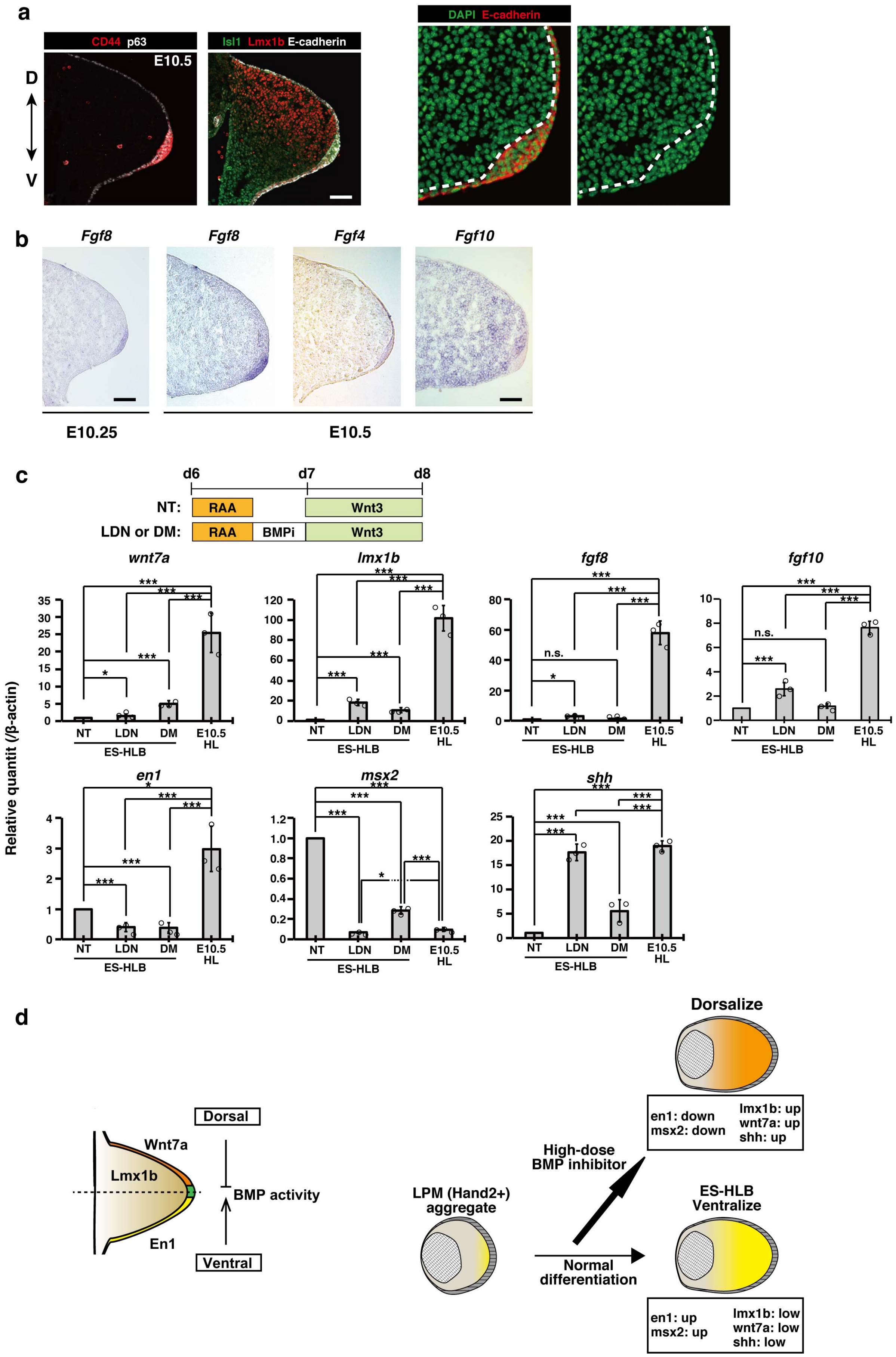
a
CD44 p63
E10.5
Isl1 Lmx1b E-cadherin
DAPI E-cadherin
D
V
b
Fgf8
Fgf8
Fgf4
Fgf10
E10.25
E10.5
c
d6
d7
d8
NT:
RAA
Wnt3
LDN or DM:
RAA
BMPi
Wnt3
wnt7a
lmx1b
fgf8
fgf10
en1
msx2
shh
Relative quantit (/β-actin)
NT
LDN
DM
E10.5 HL
ES-HLB
n.s.
d
Dorsal
Wnt7a
Lmx1b
BMP activity
En1
Ventral
LPM (Hand2+) aggregate
High-dose BMP inhibitor
Normal differentiation
Dorsalize
en1: down
msx2: down
lmx1b: up
wnt7a: up
shh: up
ES-HLB
Ventralize
en1: up
msx2: up
lmx1b: low
wnt7a: low
shh: low

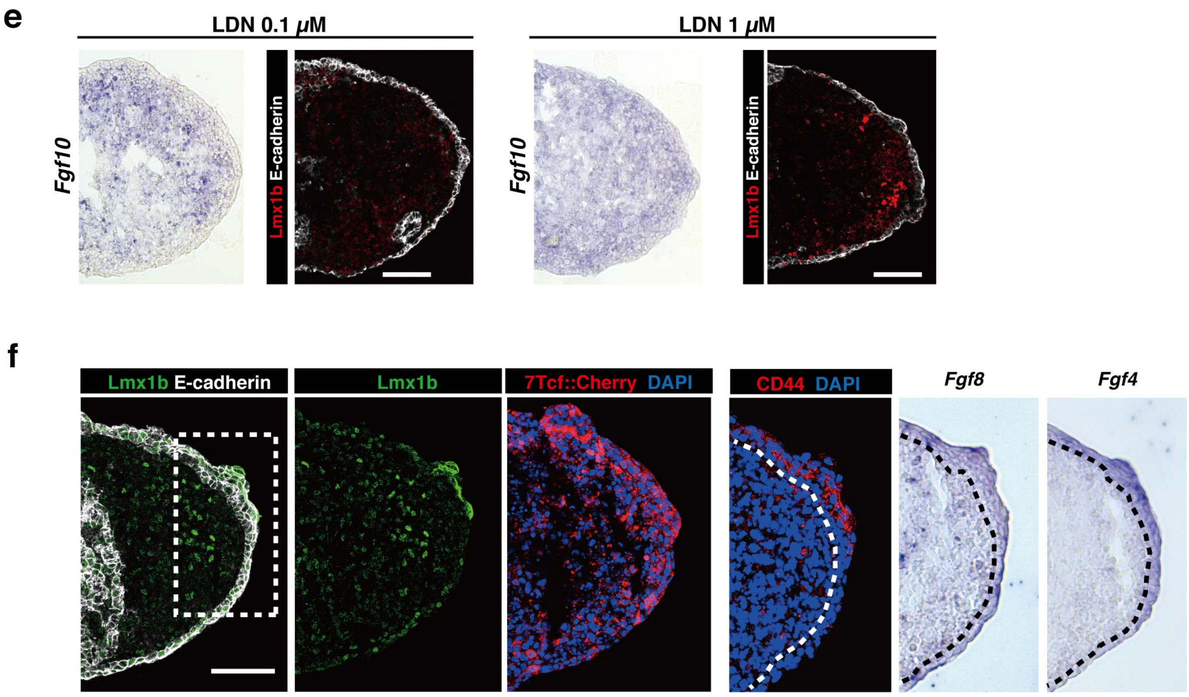

Supplementary Figure 9 BMP inhibitors induce dorsalization in the ES-LB. (**a, b**) Transverse sections of the mouse hindlimb bud. Immunostaining of AER marker (CD44) and D-V mesenchymal markers (D, Lmx1b; V, Isl1) (**a**, left). Enlarged view of AER (E-cadherin+ thickness ectodermal area; **a**, right). White dotted line indicates a boundary between ectoderm and mesenchyme. (**b**) Section *in situ* hybridization of *Fgf4, 8, 10* in hindlimb bud. *Fgf4* and *8* are expressed in AER, and *Fgf10* is expressed in limb mesenchyme. (**c**) Experimental timeline showing treatment period of BMP inhibitors (top). qRT-PCR analysis of the expression of limb bud dorsal (*wnt7a, lmx1b*) and ventral (*msx1, en1*) markers in whole ES-HLB by adding high-dose LDN and DM (mean ± s.d, n=3 independent experiments, *P < 0.05, **P < 0.01, ***P < 0.005 compared to NT; two-tailed Student's t-test, bottom graphs). (**d**) Schematic model of the limb bud (right) and the ES-HLB (left) dorsal-ventral polarity. According to an in vivo BMP antagonist effect, the treatment of BMP inhibitors (LDN and DM) induce a dorsalization in the ES-HLB. (**e**) *in situ* hybridization of *Fgf10* and immunostaining for Lmx1b and E-cadherin in the 0.1 and 1 μM LDN-treated ES-HLB on day 9. (**f**) Cryosection of the LB-region of 1 μM LDN-treated ES-HLB on day 9. LDN induced the expression of Lmx1b in ES-LB mesenchyme. *7Tcf::Cherry* activity detecting in the surface ectoderm. Random thickened ectoderm expresses AER markers (CD44+, *Fgf8*+, *Fgf4*+, high-magnification view of dotted box area in the left panel). Dotted line indicates a boundary between ectoderm and mesenchyme. NT, non-treat; LDN, LDN193189; DM, Dorsomorphin. Scale bars, 100 μm.

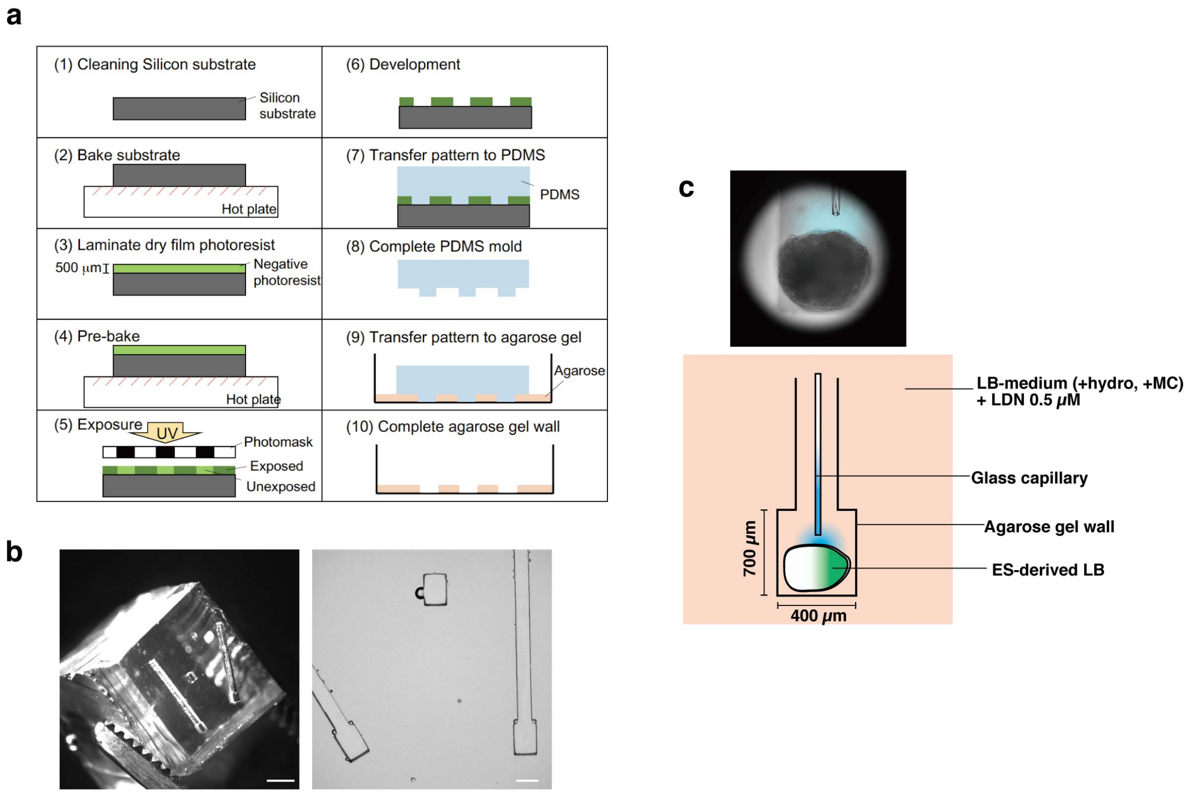

Supplementary Figure 10 Fabrication of an agarose wall for local application system of the ES-LB. (**a**) Photolithography process of the PDMS gel mold (ref. Methods). (**b**) PDMS gel mold (left; scale bar, 2 mm) and agarose wall (right; scale bar, 500 μm) which made by using PDMS gel mold. **c**, Snapshot of DM injected locally to the ES-LB (top) and schematic model of culture system for local application (bottom). LDN, LDN193189; DM, dorsomorphin; MC, methylcellulose; PDMS, Polydimethylsiloxane; LB-medium, limb bud-differentiation medium.

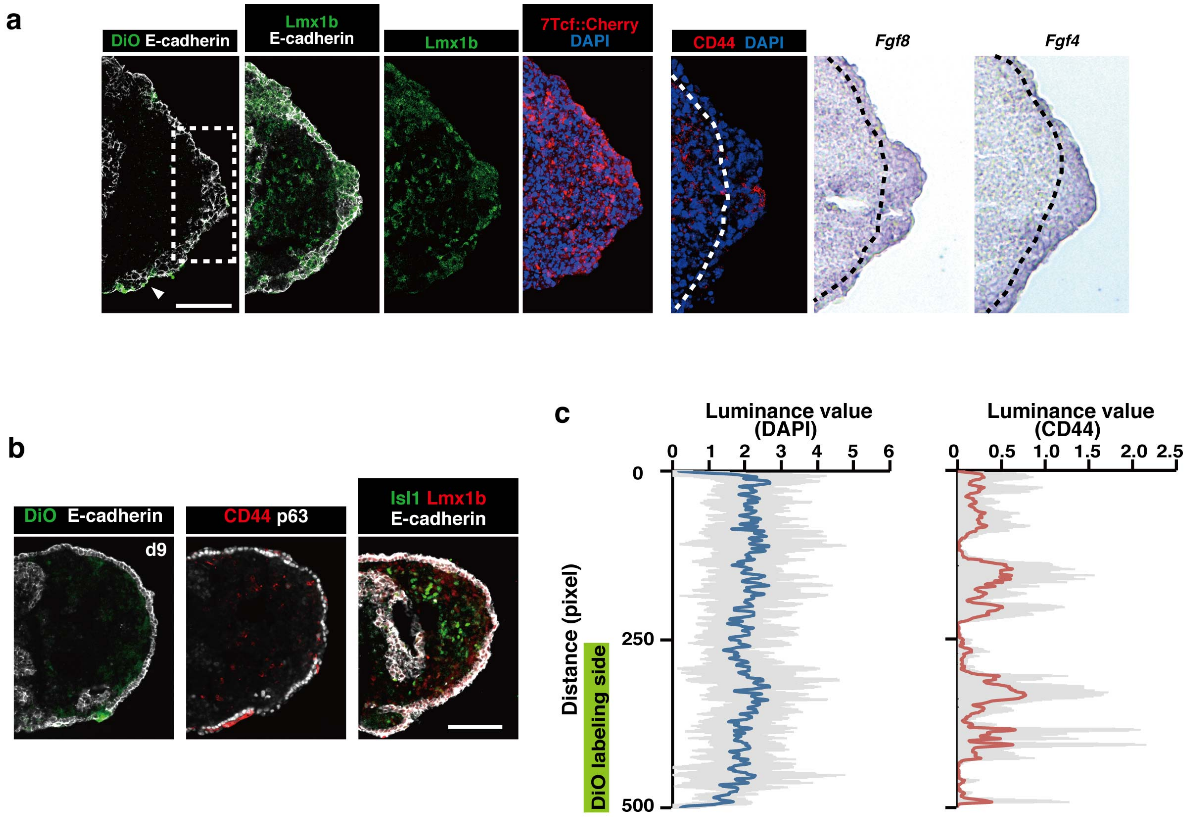

Supplementary Figure 11 Local-injection of DM induces AER-like tissue in the ES-LB ectoderm. (**a**) Cryosection of locally DM-injected ES-HLB. The injected side was labeled by DiO (arrowhead). LDN induced the expression of Lmx1b in ES-LB mesenchyme. *7Tcf::Cherry* activity detecting in the surface ectoderm. Thickened ectoderm formed at the tip of ectoderm, and expresses AER markers (CD44+, *Fgf8*+, *Fgf4*+, high-magnification view of dotted box area in the left panel). Dotted line indicates a boundary between ectoderm and mesenchyme.. Local injection of LDN does not induce the AER-like structure. (**b**) Cryosection of locally LDN-injected ES-HLB. Lmx1b and Isl1 use as D-V marker. Injected side was labeled by DiO. CD44 and p63 use as AER and epidermis marker, respectively. (**c**) Quantitative analysis in a thickness of the epithelial layer (left, blue line) and CD44-expressed region (right, red line) in locally LDN-injected ES-HLB (light gray, mean ± s.d, n=6 independent experiments). Scale bars, 100 μm.

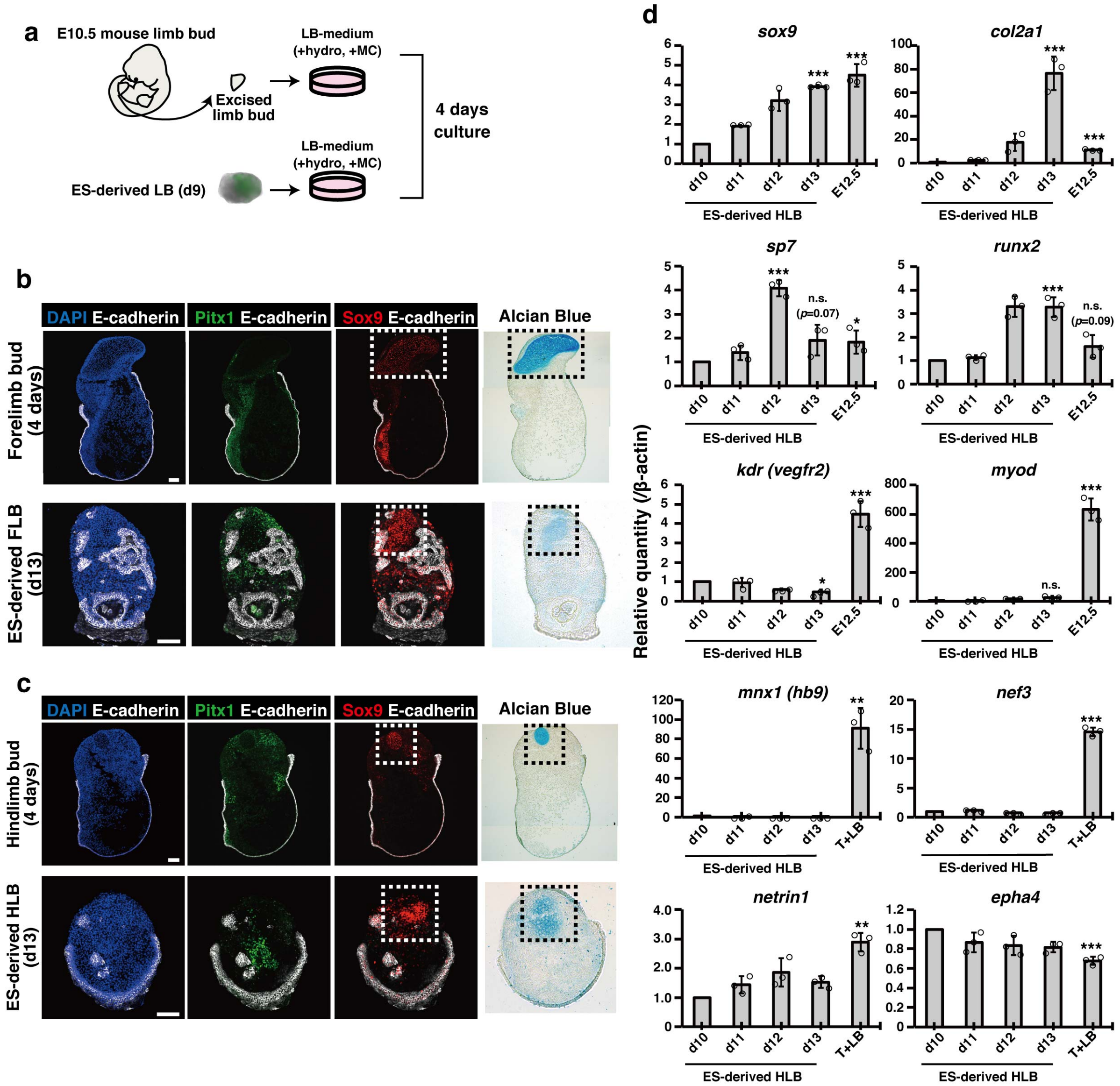

Supplementary Figure 12 Self-chondrogenesis in ES-LB. (**a**) Schematic of the in vitro culture for chondrogenesis. (**b, c**) Cryosection of the mouse limb buds and ES-LB at 4 days after culturing (**b**, forelimb; **c**, hindlimb). Mesenchymal cells overflow from ectoderm uncovering area (E-cadherin-Pitx1 or -Tbx5). Chondrocyte is stained with Sox9 and Alcian Blue indicating dotted box. Scale bar, 100 μm. (d) qRT-PCR analysis of ES-HLB from day 10 to 13, E11.5 mouse hindlimb bud and trunk tissue(T+LB), and E12.5 mouse hindlimb bud (mean ± s.d, *n*=3 independent experiments, $^*P < 0.05$, $^{**}P < 0.01$, $^{***}P < 0.005$ compared to d10 samples; two-tailed Student's *t*-test). The expression of cartilage-related genes (*sox9, sp7, runx2,* and *col2a1*) are increased in ES-LB. Angiogenesis (*kdr*)-, muscle (*myod*)-, and motor neuron (*mnx1* and *nef3*)-related genes are not sufficiently expressed, while axon guidance-related genes (*netrin1* and *epha4*) are expressed in ES-LB.

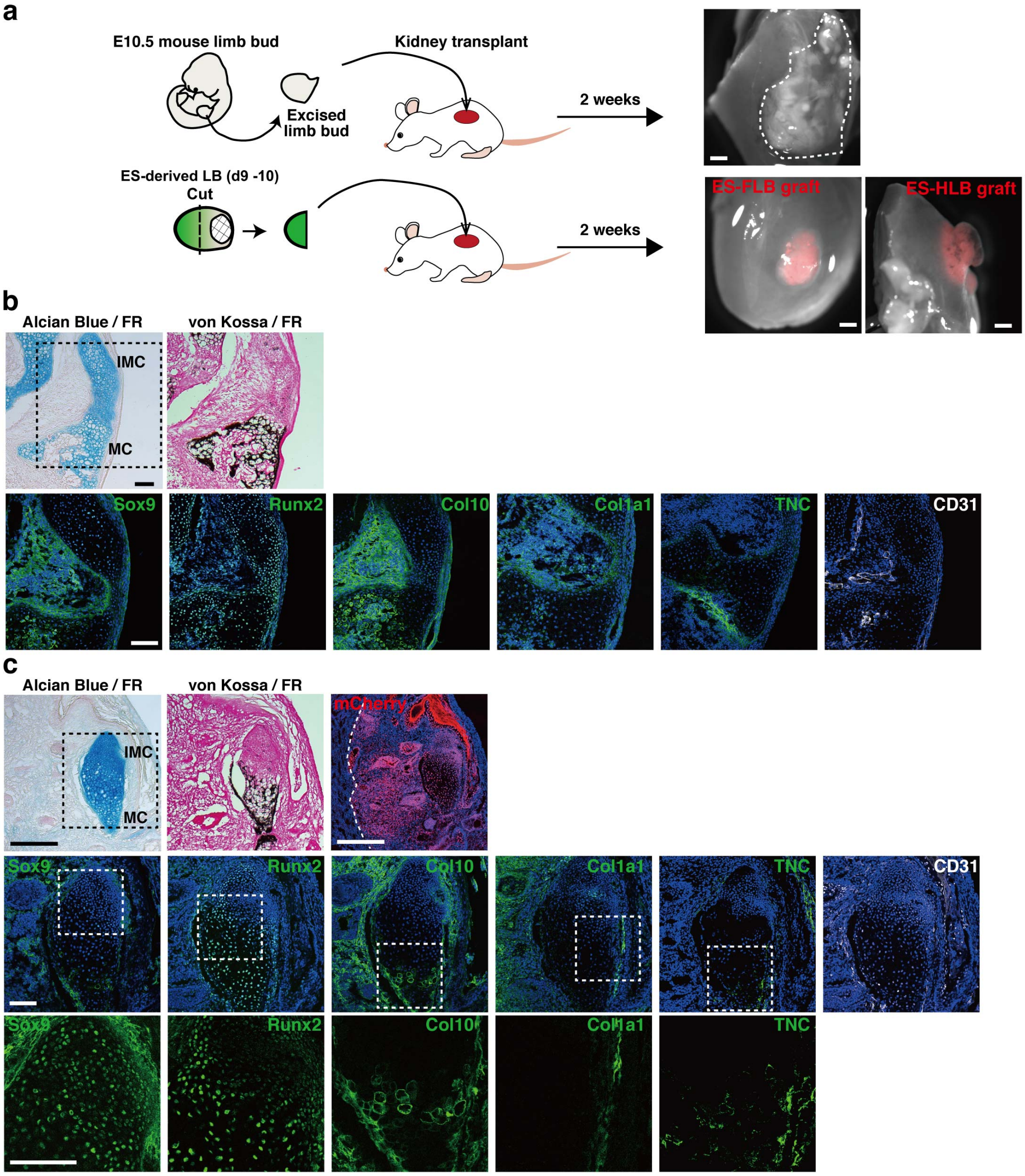

Supplementary Figure 13 Endochondral ossification of LB-like tissues under the renal capsule. (**a**) Schematic of the experiment (left), and right pictures show kidneys transplanted with mouse forelimb buds (dotted line area; top) and ES-LB (*pCAG-H2B::mCherry* line; bottom) grafts at 2 weeks post-transplantation. Scale bar, 1 mm. (**b, c**) Cryosection of grafts of mouse forelimb bud (b, *n* = 5), ES-HLB (c, *n* = 6). Alcian Blue staining indicates chondrocyte tissue, and von Kossa staining indicates calcification of cartilage. Black dotted box in Alcian blue staining panel indicates the area of immunostating of osteogenesis markers. White dotted lines indicate a boundary

between host and mCherry$^+$ graft (**c**). Mouse forelimb bud and ES-HLB grafts differentiated into IMC (Sox9$^+$, Runx2$^+$), MC (von Kossa$^+$, Runx2$^+$, Col10$^+$, Col1a1$^+$) and tendon (TNC$^+$) differentiation (**b, c**). The lower pictures show higher magnifications of the white dotted box areas in the each of upper pictures (c). Blood vessels form around the each of osteogenesis area (CD31$^+$; platelet/endothelial cell adhesion molecule-1). IMC, immature chondrocyte; MC, mature chondrocyte; FR, nuclear Fast Red; Col10, type X collagen; Col1a1, type 1a1 collagen; TNC, tenascin C. Scale bars, 100 μm.

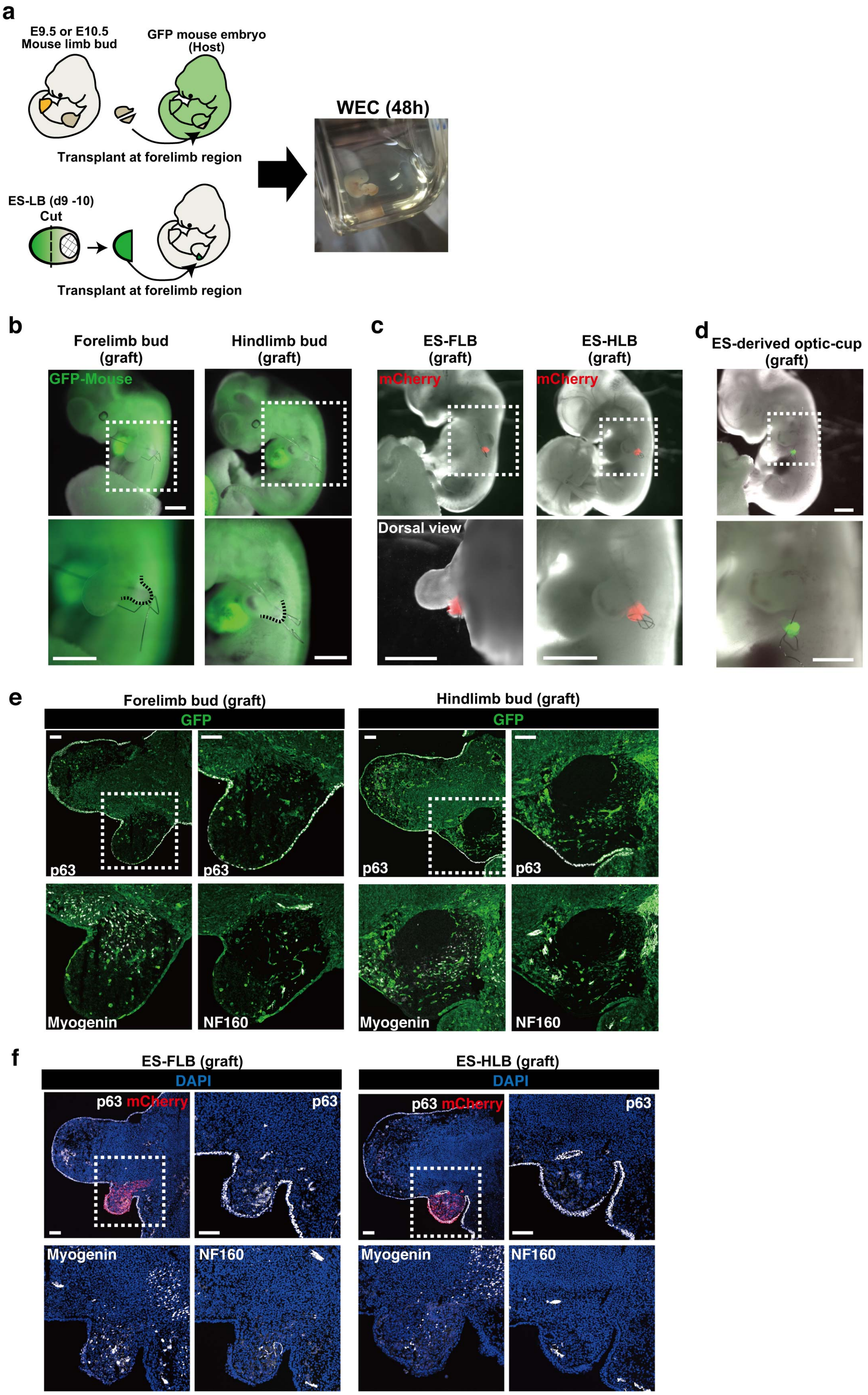
a
E9.5 or E10.5
Mouse limb bud
GFP mouse embryo
(Host)
Transplant at forelimb region
ES-LB (d9 -10)
Cut
Transplant at forelimb region
WEC (48h)
b
Forelimb bud
(graft)
Hindlimb bud
(graft)
GFP-Mouse
c
ES-FLB
(graft)
ES-HLB
(graft)
mCherry
mCherry
Dorsal view
d
ES-derived optic-cup
(graft)
e
Forelimb bud (graft)
Hindlimb bud (graft)
GFP
GFP
p63
p63
p63
p63
Myogenin
NF160
Myogenin
NF160
f
ES-FLB (graft)
ES-HLB (graft)
DAPI
DAPI
p63 mCherry
p63
p63 mCherry
p63
Myogenin
NF160
Myogenin
NF160

Supplementary Figure 14 Myoblast migration and neural projection into the ES-LB. (**a**) Schematic of transplantation with WEC system. (**b-d**) Engraftment state of E9.5 mouse forelimb bud (**b**, left) and E10.5 hindlimb bud (**b**, right) graft, ES-FLB (**c**, left) and -HLB (**c**, right) graft, and ES-derived optic-cup after 48h culture. Scale bar, 1 mm. Mouse limb bud grafts showing black dotted line (non-GFP fluorescence tissue). ES-LB (*pCAG-H2B::mCherry* line) and ES-derived optic-cup grafts showing mCherry and *Rx::GFP* fluorescence, respectively. ES-derived optic-cup did not engraft into the forelimb bud region. Scale bar, 1 mm. (**e, f**) Cryosection of grafts of mouse fore (**e**, left), hind (e, right) limb bud and ES-FLB (**f**, left), and -HLB (**f**, right). Each graft connects with host ectoderm (p63+). Myoblast (myogenin+) and neuronal fiber (NF160+) innervating into grafts from host embryo. Scale bars, 100 μm.

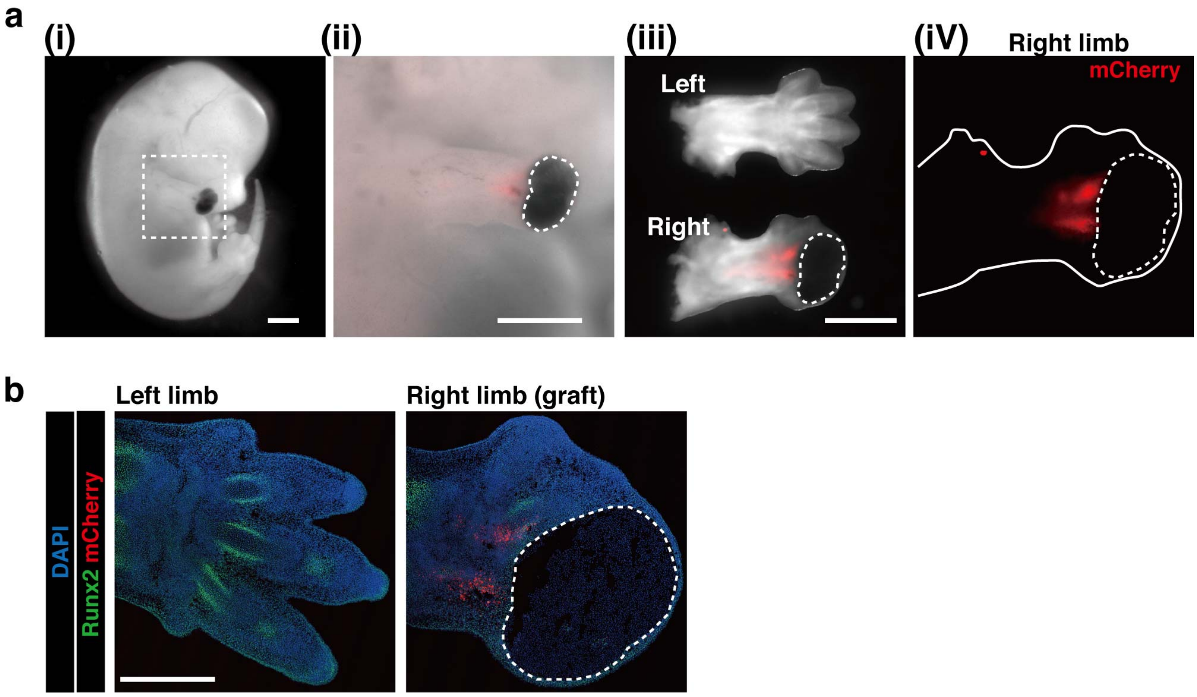

Supplementary Figure 15 mESCs-derived IM-like cells inhibit limb bud development after *exo-utero* transplantation. (**a**) 2 days post-grafting. IM-like cells (*pCAG-H2B::mCherry* line) grafted into embryonic right-forelimb bud. Panel-(ii) is enlarged view of dotted box of panel-(i). Panel-(iii) shows a comparison between non-grafted (left limb bud, upper) and grafted (right limb bud, lower) forelimb bud. Panel-(iv) shows enlarged view of mCherry^{+} transplant site in right-forelimb bud. Dotted circles indicate the blood clot. Scale bars, 1mm. (**b**) Immunostaining of sagittal section of the non-grafted and grafted limb bud. mESCs-derived IM-like cells (mCherry^{+}) inhibit a digit formation (Runx2^{+}) by forming the blood clot (dotted circle, *n*=2). Scale bars, 500 μm.

Supplementary Table 1 Ratio of engraftment and trunk-derived cells migration in WEC transplantation.

The rate of engraftment at mouse fore-lim bud region

	Total	Epidrmis connection	Myoblast	Nueron	Sox9+ cells accumulation
ES-derived FL	6	4/6 (66.7%)	4	4	-
ES-derived HL	5	4/5 (80%)	4	4	-
mFLB (E9.5)	5	4/5 (80%)	3	2	-
mFLB (E10.5)	2	2/2 (100%)	2	2	2
mHLB (E10.5)	7	2/7 (28.6%)	1	1	1
ES-derived optic cup	3	0/3 (0%)	-	-	-

Epidermis connection showing the rate of engraftment. Among the allograft, the number of graft which observed migration of myoblast and neuronal fiber and chondrogenesis shown. mFLB; mouse forelimb bud, mHLB; mouse hindlimb bud.
