# Supplementary material for: Self-organized formation of developing appendages from murine pluripotent stem cells
Source: Nat Commun. 2019 Aug 23;10:3802. doi: 10.1038/s41467-019-11702-y (PMC6707191; doi:10.1038/s41467-019-11702-y)
Supplement: Supplementary file 7 — Description of Additional Supplementary Files [file 41467_2019_11702_MOESM7_ESM.docx]

**Title: Supplementary Movie 1**

**Description:** ESC-aggregate differentiates into PPS-aggregate. Live imaging of mESC-aggregate (*7Tcf::Cherry* line) from day 3.5 to 5. *7Tcf::Cherry* positive cells differentiate in a polarized manner in the inner cell aggregate from day 4. Left and right movie indicate the single image of *7Tcf::Cherry* (Red) and the marge of *7Tcf::Cherry* (Red) and bright filed view, respectively.

**Title: Supplementary Movie 2**

**Description:** The removal of amniotic-like membrane from PPS-aggregate.

Live imaging of the removing process of an amniotic-like membrane which wraps a PPS-aggregate on day 5.

**Title: Supplementary Movie 3**

**Description:** 3D-reconstructed movie of ES-HLB on day 9.

Whole immunostaining (Green, PDGFR; Red, E-cadherin) of ES-HLB. Mesenchymal cells aggregate (Green) is covered by the epidermis layer (Red).

**Title: Supplementary Movie 4**

**Description:** 3D-reconstructed ES-HLB in bath-application of DM.

Whole immunostaining of CD44 (Cyan) and E-cadherin (Gray). In bath-application of DM, thickness epidermis (CD44+) forms randomly.

**Title: Supplementary Movie 5**

**Description:** 3D-reconstructed ES-HLB in local-application of DM.

Whole immunostaining of CD44 (Cyan) and E-cadherin (Gray). Local-application of DM induce the thickness epidermis (CD44^+^) at the tip of ES-HLB.
