# Supplementary material for: Self-organized formation of developing appendages from murine pluripotent stem cells
Source: Nat Commun. 2019 Aug 23;10:3802. doi: 10.1038/s41467-019-11702-y (PMC6707191; doi:10.1038/s41467-019-11702-y)
Supplement: Supplementary file 8 — Reporting Summary [file 41467_2019_11702_MOESM8_ESM.pdf]

nature research

Corresponding author(s): Mototsugu Eiraku, Shunsuke Mori

☒ Initial submission ☐ Revised version ☐ Final submission

Life Sciences Reporting Summary

Nature Research wishes to improve the reproducibility of the work that we publish. This form is intended for publication with all accepted life science papers and provides structure for consistency and transparency in reporting. Every life science submission will use this form; some list items might not apply to an individual manuscript, but all fields must be completed for clarity.

For further information on the points included in this form, see Reporting Life Sciences Research. For further information on Nature Research policies, including our data availability policy, see Authors & Referees and the Editorial Policy Checklist.

Experimental design

1. Sample size

Describe how sample size was determined.	Sample sizes were chosen based on preliminary experiments so as to provide sufficient values for statistical comparison.

2. Data exclusions

Describe any data exclusions.	no data exclusion

3. Replication

Describe whether the experimental findings were reliably reproduced.	All the repilications is reported in the manuscript.

4. Randomization

Describe how samples/organisms/participants were allocated into experimental groups.	n/a

5. Blinding

Describe whether the investigators were blinded to group allocation during data collection and/or analysis.	n/a

Note: all studies involving animals and/or human research participants must disclose whether blinding and randomization were used.

6. Statistical parameters

For all figures and tables that use statistical methods, confirm that the following items are present in relevant figure legends (or in the Methods section if additional space is needed).

n/a	Confirmed	
☐	☒	The exact sample size (n) for each experimental group/condition, given as a discrete number and unit of measurement (animals, litters, cultures, etc.)
☐	☒	A description of how samples were collected, noting whether measurements were taken from distinct samples or whether the same sample was measured repeatedly
☐	☒	A statement indicating how many times each experiment was replicated
☐	☒	The statistical test(s) used and whether they are one- or two-sided (note: only common tests should be described solely by name; more complex techniques should be described in the Methods section)
☒	☐	A description of any assumptions or corrections, such as an adjustment for multiple comparisons
☐	☒	The test results (e.g. P values) given as exact values whenever possible and with confidence intervals noted
☐	☒	A clear description of statistics including central tendency (e.g. median, mean) and variation (e.g. standard deviation, interquartile range)
☐	☒	Clearly defined error bars

See the web collection on statistics for biologists for further resources and guidance.

June 2017

Software

Policy information about availability of computer code

7. Software

Describe the software used to analyze the data in this study.	Softwares used to analyze the data are reported in the manuscript.

For manuscripts utilizing custom algorithms or software that are central to the paper but not yet described in the published literature, software must be made available to editors and reviewers upon request. We strongly encourage code deposition in a community repository (e.g. GitHub). *Nature Methods* guidance for providing algorithms and software for publication provides further information on this topic.

Materials and reagents

Policy information about availability of materials

8. Materials availability

Indicate whether there are restrictions on availability of unique materials or if these materials are only available for distribution by a for-profit company.	Antibodies for Pitx1, Tbx5, Irx3 were custom made by Takara Bio inc.<br>Antibody for Lmx1b was custom made by IBL Co., Ltd., Japan.

9. Antibodies

Describe the antibodies used and how they were validated for use in the system under study (i.e. assay and species).	The specificity was confirmed by comparing the staining of the mouse section with the known expression pattern.

10. Eukaryotic cell lines

a. State the source of each eukaryotic cell line used.	Mouse embryonic stem cell lines:<br>EB5 (RIKEN BRC, Cell No. AES0151)<br>E14tg2a (RIKEN BRC, Cell No. AES0135)
b. Describe the method of cell line authentication used.	The method is reported in the manuscript.
c. Report whether the cell lines were tested for mycoplasma contamination.	Tested
d. If any of the cell lines used are listed in the database of commonly misidentified cell lines maintained by ICLAC, provide a scientific rationale for their use.	n/a

Animals and human research participants

Policy information about studies involving animals; when reporting animal research, follow the ARRIVE guidelines

11. Description of research animals

Provide details on animals and/or animal-derived materials used in the study.	For the whole embryo culture, we used ICR and C57BL/6-Tg (CAG-EGFP) mouse lines. These were obtained from Japan SLC, Inc.

Policy information about studies involving human research participants

12. Description of human research participants

Describe the covariate-relevant population characteristics of the human research participants.	n/a

nature research

Corresponding author(s): Motosugu Eiraku, Shunsuke Mori

☐ Initial submission ☒ Revised version ☐ Final submission

Flow Cytometry Reporting Summary

Form fields will expand as needed. Please do not leave fields blank.

Data presentation

For all flow cytometry data, confirm that:

☒ 1. The axis labels state the marker and fluorochrome used (e.g. CD4-FITC).

☒ 2. The axis scales are clearly visible. Include numbers along axes only for bottom left plot of group (a 'group' is an analysis of identical markers).

☒ 3. All plots are contour plots with outliers or pseudocolor plots.

☒ 4. A numerical value for number of cells or percentage (with statistics) is provided.

Methodological details

5. Describe the sample preparation.	The method is reported in the manuscript.
6. Identify the instrument used for data collection.	All data was analyzed using BD FACS AriaIII (BD Bioscience)
7. Describe the software used to collect and analyze the flow cytometry data.	FlowJo v10
8. Describe the abundance of the relevant cell populations within post-sort fractions.	The abundance cell population of Day5 cell aggregates (posterior primitive streak): average 3.4 x 10^5 cells.<br>The abundance cell population of Day9 cell aggregates (hindlimb bud-like tissue): average 6.3 x 10^5 cells.<br>The abundance cell population of Day9 cell aggregates (forelimb bud-like tissue): average 7.6 x 10^5 cells.<br>The purity is indicated in the manuscript.
9. Describe the gating strategy used.	The starting cell population collected by FSC/SSC gate. In the next gate, these cells were sorted by YG-PE-A (7Tcf::cherry) or FITC-A (Hand2::mEGFP) positive cell population which gates were created by comparison data of control cell range (ES cell or undifferentiated cell).

Tick this box to confirm that a figure exemplifying the gating strategy is provided in the Supplementary Information. ☒
